# Biochemical and biophysical drivers of the hydrogen isotopic composition of carbohydrates and acetogenic lipids

**DOI:** 10.1126/sciadv.adl3591

**Published:** 2024-07-10

**Authors:** Marco M. Lehmann, Philipp Schuler, Roland A. Werner, Matthias Saurer, Guido L. B. Wiesenberg, Marc-André Cormier

**Affiliations:** ^1^ Forest Dynamics, Swiss Federal Institute for Forest, Snow and Landscape Research WSL, Zuercherstrasse 111, 8903 Birmensdorf, Switzerland.; ^2^ Forest Soils and Biogeochemistry, Swiss Federal Institute for Forest, Snow and Landscape Research WSL, Zuercherstrasse 111, 8903 Birmensdorf, Switzerland.; ^3^ School of Architecture, Civil and Environmental Engineering, École Polytechnique Fédérale de Lausanne, Lausanne, Switzerland.; ^4^ D-USYS–Department of Environmental Systems Science, ETH Zurich, Universitaetstrasse 2, 8092 Zurich, Switzerland.; ^5^ Department of Geography, University of Zurich, Winterthurerstrasse 190, 8057 Zurich, Switzerland.; ^6^ Department of Earth Sciences, University of Oxford, South Parks Road, Oxford OX1 3AN, UK.

## Abstract

The hydrogen isotopic composition (δ^2^H) of plant compounds is increasingly used as a hydroclimatic proxy; however, the interpretation of δ^2^H values is hampered by potential coeffecting biochemical and biophysical processes. Here, we studied δ^2^H values of water and carbohydrates in leaves and roots, and of leaf *n*-alkanes, in two distinct tobacco (*Nicotiana sylvestris*) experiments. Large differences in plant performance and biochemistry resulted from (a) soil fertilization with varying nitrogen (N) species ratios and (b) knockout-induced starch deficiency. We observed a strong ^2^H-enrichment in sugars and starch with a decreasing performance induced by increasing NO_3_^−^/NH_4_^+^ ratios and starch deficiency, as well as from leaves to roots. However, δ^2^H values of cellulose and *n*-alkanes were less affected. We show that relative concentrations of sugars and starch, interlinked with leaf gas exchange, shape δ^2^H values of carbohydrates. We thus provide insights into drivers of hydrogen isotopic composition of plant compounds and into the mechanistic modeling of plant cellulose δ^2^H values.

## Teaser

Strong hydrogen isotope fractionation in carbohydrates reveals underlying mechanisms driving δ^2^H values in organic compounds.

## INTRODUCTION

Hydrogen isotope ratios (δ^2^H) measured on acetogenic lipids (e.g., *n*-alkanes) and carbohydrates (e.g., cellulose) extracted from plant tissues of aquatic or terrestrial archives are widely applied for studying past hydroclimatic conditions (*1*–*4*). A growing body of evidence suggests that δ^2^H values of carbohydrates and lipids may integrate not only (eco-)climatic but also biophysical and biochemical information (*5*–*9*). Such nonclimatic information may hinder the reconstruction of climatic conditions based on δ^2^H values and urges for a better understanding of the key drivers of δ^2^H values in plant compounds. However, once disentangled, the nonclimatic information in δ^2^H values may open avenues for an improved mechanistic understanding of plant-climate interactions.

δ^2^H values of plant compounds are initially determined by the source water, which is defined as the water taken up by roots and transported in the xylem stream without isotopic fractionations (*10*). In leaves, the source water is mixed with atmospheric water vapor and enriched in heavier water isotopes due to the evaporative loss of lighter isotopologs (*11*–*13*). Source and leaf water are fundamental for the δ^2^H values of plant organic compounds, with the relative importance of both water pools depending on whether compounds are produced in autotrophic (e.g., leaves) or heterotrophic (e.g., stems and roots) tissues (*14*, *15*). Previous studies suggested that the ^2^H-fractionation between the water pools and compounds in plants is constant (*14*–*19*), similar as for δ^18^O (*20*), enabling temperature reconstructions at continental or global scale (*1*, *21*–*23*). However, the sensitivity for climatic signals has been found to be much higher for δ^18^O compared to δ^2^H values in tree-ring cellulose (*24*), highlighting that the ^2^H-fractionation in plant carbohydrates is more dynamic than previously anticipated. This is supported by studies observing δ^2^H variations of plant compounds independent of the water signal with environmental factors (*7*, *25*–*27*), site conditions (*24*, *28*), nonclimatic leaf defoliator outbreaks (*29*), and leaf development (*8*, *30*). While the key biochemical and biophysical drivers causing these δ^2^H variations in plant compounds are not well understood, a recent study observed that carbohydrate δ^2^H values are likely more dynamic than lipids (*9*).

### Biophysical considerations

δ^2^H values in plant compounds differ among plants with different photosynthetic pathways (*31*–*33*), suggesting that δ^2^H values are shaped by photosynthetic processes and leaf gas exchange. For instance, δ^2^H values of plant carbohydrates and *n*-alkanes in grass or herbaceous species become ^2^H-enriched under low light and ambient CO_2_ concentrations (*7*), with decreasing intercellular CO_2_ concentration (*34*), and reduced assimilation and electron transport rates (*6*, *35*). Other studies observed positive relationships between δ^2^H values in plant carbohydrates and respiration rates (*6*, *36*). On the basis of negative relationships with wheat biomass production, a study suggested that δ^2^H values in plant organic matter could function as a proxy for plant performance (*35*). However, biophysical processes shaping that δ^2^H values in plant compounds have so far not been investigated systemically and are intertwined with complex biochemical processes.

### Biochemical considerations

δ^2^H variations in plant compounds are likely induced by integration of different H sources and changes in metabolic pathways (*5*, *7*, *37*, *38*). A first key compound and direct source of H atoms in organic compounds is the coenzyme nicotinamide adenine dinucleotide phosphate in its reduced form (NADPH). In the chloroplast, NADPH can be produced during the photosynthetic photolysis reaction of water by the enzyme (ferredoxin-NADP^+^ reductase, EC 1.18.1.2). The hydride transferred to NADPH is ^2^H-depleted and considered responsible for the large ^2^H-fractionation observed during autotrophic biosynthesis of plant organic matter (*7*, *14*, *32*, *39*, *40*). NADPH can also be produced by the tricarboxylic acid cycle (cytosolic NADP-dependent isocitrate dehydrogenase involved in ammonia assimilation, EC 1.1.1.42), the malate valve [responsible for redox homeostasis, oxalacetate as precursor of aspartate and other amino acids (AA)], and the oxidative pentose phosphate pathway [glucose-6-phosphate dehydrogenase (EC 1.1.1.49) and 6-phosphogluconate dehydrogenase (EC 1.1.1.44)]. These pathways are expected to produce ^2^H-enriched NADPH compared to the photosynthetical pathway (*5*, *41*, *42*). Given that acetogenic lipids are mostly produced in the chloroplast under illumination and that about 50% of their carbon-bound H atoms are derived from NADPH, this results in typically ^2^H-depleted acetogenic lipids (*7*, *9*). Structural carbohydrates, like cellulose, are primarily synthesized in the cytosol. Only 15% of the carbon bound hydrogen atoms in these carbohydrates are derived from NADPH, making them typically less ^2^H-depleted compared to acetogenic lipids.

Surrounding water can function as a second biochemical source of H atoms in organic matter, which can be integrated via hydration reactions (i.e., addition of hydrogen atoms of water) and tautomerization (i.e., H atom exchange during ketone-enol tautomerism) catalyzed by, e.g., isomerase reactions (*6*, *7*, *15*, *26*, *43*). Fresh assimilates in leaves such as sugars and transitory starch are typically synthesized in leaf water (*44*). For instance, these processes, during cellulose synthesis, can overwrite 31 to 40% of the initial leaf-level isotope signal in plant leaf assimilates (*14*, *15*, *43*, *45*) and cause ^2^H-enrichment of cellulose compared to sugars and starch in leaves (*31*).

A third biochemical source of H atoms are the metabolic precursor molecules that provide the initial carbon skeletons for carbohydrates [ribulose 1,5-bisphosphate in the Calvin-Benson Bassham (CBB) cycle] and acetogenic lipids [acetyl–coenzyme A (CoA)] which typically reflect their photosynthetic and/or glycolytic origin. Such precursors can be derived from not only fresh assimilates but also from short- or long-term storage compounds and may consequently integrate different isotopic signals. Recent studies hypothesized that relative changes in sugar and starch concentrations, implying variable turnover times, might change the likelihood of H isotope exchange with leaf water and thus cause δ^2^H variations in plant carbohydrates (*7*). A recent study provide evidence that an increase in sugar concentrations within the total nonstructural carbohydrate (NSC) pool leads to a ^2^H-enrichment in leaf sucrose across different species (*6*). This concept finds backing in a study that assessed the intramolecular hydrogen isotope patterns in glucose obtained from cellulose within tree rings (*26*). This study indicates that fluctuations in NSCs at the leaf level affect the hydrogen isotopic composition of plant assimilates, which, in turn, influences the hydrogen isotopic composition of tree-ring cellulose.

Furthermore, transitory starch in leaf chloroplasts of C3 plants has been found to be ^2^H-depleted compared to cytosolic sugars and cellulose by approximately 30 and 60‰, respectively (*31*). This is believed to mainly result from the kinetic ^2^H isotope effect on the phosphoglucose isomerase (EC 5.3.1.9) in plastid-catalyzed reaction (*31*, *46*). In contrast, storage compounds in heterotrophic tissues (e.g., branches, stems, and roots) are suggested to be ^2^H-enriched compared to those in leaves (*25*, *30*, *35*, *47*). Thus, in the case of precursors derived from heterotrophic storage compounds in higher proportion than from fresh assimilates, this could lead to ^2^H-enrichment in certain plant compounds (*28*). However, actual measurements of δ^2^H values of sugars and starch in heterotrophic tissues are lacking.

The main aim of this study was to investigate how relative changes in biochemical and biophysical traits influence ^2^H-fractionation in the biosynthesis of carbohydrates and acetogenic lipids. We therefore took advantage of two growth chamber experiments performed on tobacco plants (*Nicotiana sylvestris*). Both experiments are described to cause substantial changes in leaf gas exchange (e.g., assimilate rates and stomatal conductance) and assimilate pools (e.g., sugar and starch concentrations). In the first experiment (*48*), tobacco plants were treated with 6 mM N fertilization solutions differing in their nitrate to ammonium ratio (NO_3_^−^/NH_4_^+^) with plants under NO_3_^−^- performing better than under NH_4_^+^-dominated conditions (e.g., higher assimilation rates, starch concentrations, and biomass) (figs. S1 and S2). In the second experiment (*49*), the effects of starch deficiency in phosphoglucomutase (*pgm*) knockout mutant compared to wild-type (WT) plants were explored with WT plants performing better than *pgm* knockout plants. The *pgm* mutant reacts to the lack of leaf starch by an increased production of sugars compared to WT plants. Applying an improved water vapor equilibration method (*31*), we determined the nonexchangeable hydrogen isotopic composition in sugars, starch, and cellulose. We then also measured the δ^2^H values of leaf *n*-alkanes and water in the biomass produced in these experiments. Our main findings are as follows:

1) that δ^2^H values of primary assimilates are more sensitive than those of cellulose and *n*-alkanes to both the N fertilization treatment and starch deficiency;

2) that biophysical and biochemical traits severely affect δ^2^H values of sugars and starch, leading to a ^2^H-enrichment in carbohydrates of plants with lower performance;

3) that starch and sugars stored in heterotrophic roots are relatively ^2^H-enriched compared to those in autotrophic leaves;

4) that mechanistic models for δ^2^H of cellulose need to consider dynamic rather than constant ^2^H-fractionation factors.

### Isotope theory

Because of the complexity of all the processes causing ^2^H-fractionation in plants, we compartmentalize them into two apparent isotope fractionation factors following previous publications on the topic (*6*, *14*, *15*, *17*, *40*, *50*):

1) The apparent autotrophic ^2^H-fractionation factor (ε_a_) of a compound reflects the total of ^2^H-fractionations occurring between a compound and leaf water during the biosynthesis of the compound. More specifically, ε_a_ is an estimate that reflects the total of all isotope fractionation processes that occur during the biosynthesis of plant compounds in leaves, including, e.g., the biosynthesis of triose phosphate in the CBB cycle and reactions downstream in biochemical pathways like sugar-starch partitioning and biosynthesis of hexoses, sucrose, and lipids. The major purpose of ε_a_ is to normalize the isotopic composition of leaf compounds for leaf water isotope variations to facilitate comparison across treatments. It is typically defined as the δ^2^H difference between water and compounds in leaves (*7*)

$$\varepsilon_{a}=\delta^{2}H_{\text{Compound}}-\delta^{2}H_{\text{Leaf water}}$$ (1)

2) The apparent heterotrophic ^2^H-fractionation factor (ε_h_) reflects the total of ^2^H-fractionations occurring between source and sink carbohydrates (e.g., sugar or starch versus cellulose or leaf compounds versus root compounds). For completion, we also estimated the isotope difference between sugars and starch in leaves. Here, we defined ε_h_ as the δ^2^H difference between two carbohydrates (i.e., A or B = sugars, starch, and cellulose) within and between leaf and root tissues

$$\varepsilon_{h}=\delta^{2}H_{\text{Carbohydrate}\;A}-\delta^{2}H_{\text{Carbohydrate}\;B}$$ (2)

Specifically, we differentiate ε_h_ values between cellulose and sugars (C-S), cellulose and starch (C-St), and starch and sugars (St-S) within roots (R) and leaves (L) or between roots and leaves (R-L).

We further applied a mechanistic isotope model [i.e., Roden-Ehleringer or RE model (*15*)]. The model assumes that the δ^2^H value of cellulose is influenced by the sum of two pools: (i) the proportion of sugar or sugar phosphates (e.g., triose phosphates) that experienced autotrophic ^2^H-fractionations (ε_a*_) and are imported from the leaves to sink cells and (ii) the proportion of leaf sugars that experienced heterotrophic ^2^H-fractionations (ε_h*_) and H isotope exchange with source water before cellulose synthesis (*f*).

$$\begin{array}{c} \delta^{2}H_{\text{Cellulose}}=\left(1-f\right)*\left(\delta^{2}H_{\text{LeafWater}}+\varepsilon_{a^{*}}\right)+f\\ {}*\left(\delta^{2}H_{\text{SourceWater}}+\varepsilon_{h^{*}}\right) \end{array}$$ (3)

δ^2^H_Cellulose_, δ^2^H_LeafWater,_ and δ^2^H_SourceWater_ are the measured δ^2^H values of cellulose, leaf water, and root water, respectively. ε_a*_ and ε_h*_ reflect the constant theoretical autotrophic and heterotrophic fractionation factors of −171 and +158‰, respectively (*14*, *51*). Following results under controlled conditions, a constant f value of 0.36 was chosen (*52*). Because of potential methodological biases that affected δ^2^H values of root water during cryogenic vacuum extraction (fig. S3) (*53*), we also applied δ^2^H of soil water as δ^2^H_SourceWater_ in Eq. 3. This caused the δ^2^H values of the RE model decreased, on average, by 2.5 ‰, reflecting the maximum error for the model that could have been induced by the water extraction method.

## RESULTS

### δ^2^H values of plant compounds in response to N fertilization and tissues

**Fig. 1.**
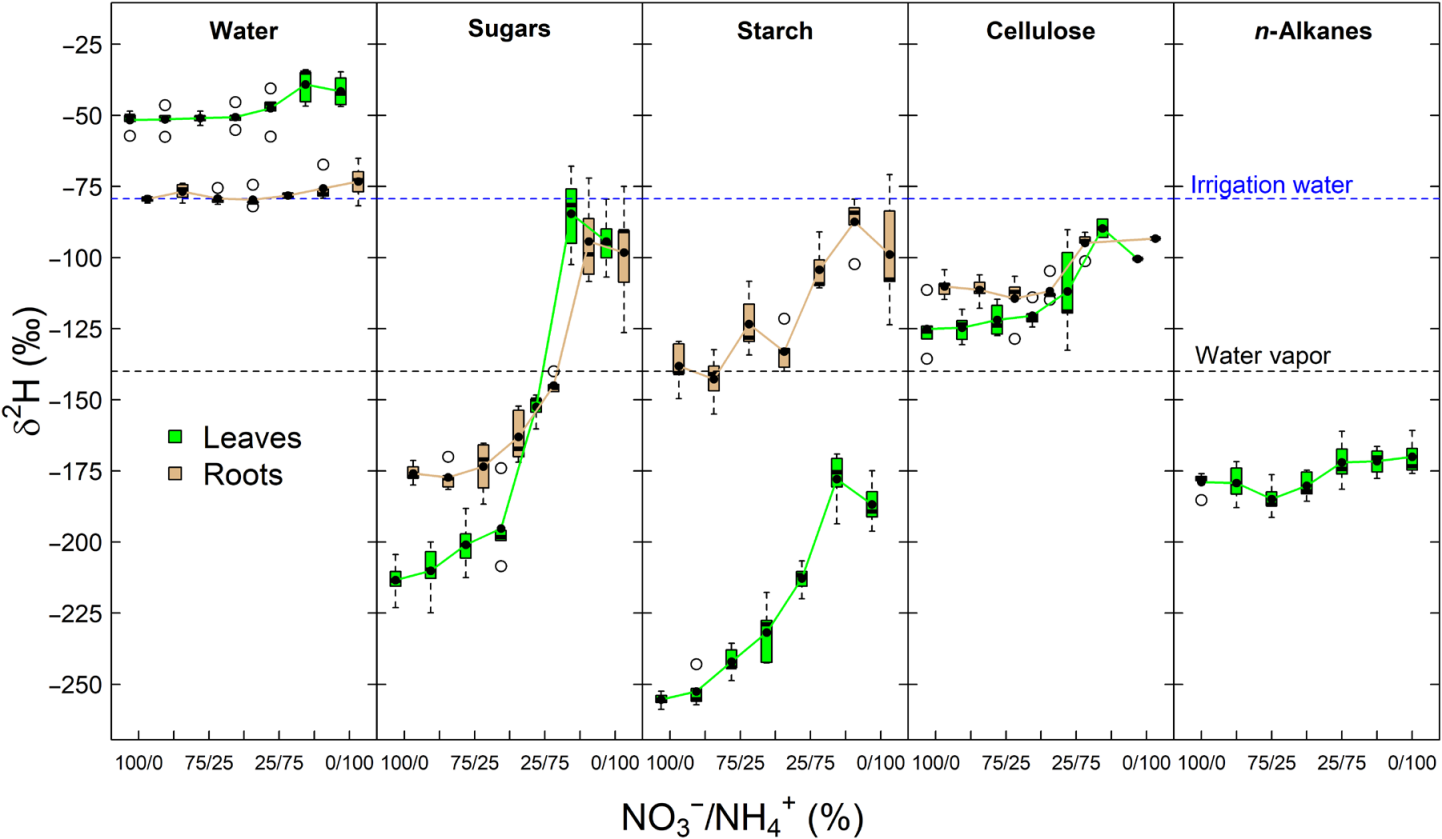
N fertilization effect on the hydrogen isotopic composition (δ^2^H) of water and compounds in tobacco (*N. sylvestris*) plants. δ^2^H values of water, carbohydrates, and *n*-alkanes in leaves and roots are shown. Plants were treated with 6 mM N fertilization solutions differing in their nitrate to ammonium ratio (NO_3_^−^/NH_4_^+^). Blue and black dashed lines denote mean δ^2^H values of irrigation water and water vapor during the experiment, respectively. δ^2^H of carbohydrates reflect the nonexchangeable carbon-bound hydrogen (δ^2^H_ne_, see Materials and Methods), while δ^2^H of *n*-alkanes represents the weighted mean of *n*-C_27_, *n*-C_29_, *n*-C_31_, and *n*-C_33_. The boxes represent the median and the 25% upper/lower quartiles, while the tails represent the 10 and 90% limits of the data.

Overall, δ^2^H values of water and plant compounds in leaves and roots were lowest under NO_3_^−^-dominated conditions (i.e., NO_3_^−^/NH_4_^+^ ratios from 100/0 to 50/50) but increased in NH_4_^+^-dominated conditions (i.e., 25/75 to 0/100). Mean δ^2^H values of water were on average 29.6‰ higher in leaves than in roots across all N fertilization treatments (*P* < 0.001) and varied by a maximum of 12.5 and 6.3‰ along the N fertilization gradient in leaves and roots, respectively (*P* < 0.001; Table 1 and Fig. 1). In contrast, sugars, starch, and cellulose were ^2^H-enriched in roots relative to leaves on average by 17.3, 104.5, and 12.3‰ across all treatments (*P* < 0.001), respectively. Mean δ^2^H values of sugars, starch, and cellulose varied by a maximum of 128.8, 77.6, and 35.4‰ in leaves and 83.0, 50.8, and 21.2‰ in roots along the N fertilization gradient (*P* < 0.001), respectively. Yet, for plant assimilates, the tissue effect was dependent on the N fertilization treatment, which was stronger in leaves than in roots (interaction N fertilization * tissue, *P* < 0.05). The N fertilization 25/75 reflect a turning point, after which δ^2^H values of assimilates in leaves and roots were higher compared to NO_3_^−^-dominated conditions but lower compared to other NH_4_^+^-dominated conditions. The weighted average mean δ^2^H value of *n*-alkanes in leaves varied by a maximum of 14.8‰ along the N fertilization gradient (*P* = 0.007).

**Table 1.** Statistical analyses of isotopic parameters. Effects of N fertilization (6 mM N fertilization solutions differing in their nitrate to ammonium ratio; NO_3_^−^/NH_4_^+^), tissue (leaves/roots), and their interactions, as well as the effect of starch deficiency (WT versus *pgm* mutant plants) on different isotope parameters in tobacco plants (*N. sylvestris*). These include hydrogen isotope (δ^2^H) values of water, carbohydrates (S, sugars; St, starch; C, cellulose) and *n*-alkanes (weighted mean of *n*-C_27_, *n*-C_29_, *n*-C_31_, and *n*-C_33_); apparent autotrophic ^2^H-fractionation factors (ε_a_); apparent heterotrophic ^2^H-fractionation factors among carbohydrates (ε_h_) differentiated between C and S (C-S), C and ST (CSt), and St and S (St-S) for roots (R), leaves (L), or between roots and leaves (R-L); and modeled δ^2^H values of cellulose (RE model, Eq. 3) according to (*15*). *P* values derived from one-way or two-way analysis of variance (ANOVA) testing the effects of N fertilization and tissue, as well as from *t* tests comparing the effect of starch deficiency. Significant results (*P* < 0.05) are marked in bold. NA, not available.

Isotope parameter	N fertilization	Tissue	N fertilization * Tissue	Starch deficiency
**δ** ^**2**^ **H water**	**<0.001**	**<0.001**	0.138	NA
**δ** ^**2**^ **H sugars**	**<0.001**	**<0.001**	**<0.001**	**<0.001**
**δ** ^**2**^ **H starch**	**<0.001**	**<0.001**	**0.016**	**<0.001**
**δ** ^**2**^ **H cellulose**	**<0.001**	**<0.001**	0.685	**<0.001**
**δ** ^**2**^ **H *n*-alkanes**	**0.007**	NA	NA	0.389
**ε**_**a**_ **Sugars**	**<0.001**	NA	NA	NA
**ε**_**a**_ **Starch**	**<0.001**	NA	NA	NA
**ε**_**a**_ **Cellulose**	**0.025**	NA	NA	NA
**ε**_**a**_ ***n*-Alkanes**	0.359	NA	NA	NA
**ε**_**h**_ **C-S (L, R, R-L)**	**<0.001**	NA	NA	NA
**ε**_**h**_ **C-St (L, R-L)**	**<0.001**	NA	NA	NA
**ε**_**h**_ **C-St (R)**	**0.009**	NA	NA	NA
**ε**_**h**_ **St-S (L, R, R-L)**	**<0.001**	NA	NA	NA
**δ** ^**2**^ **H RE model**	**<0.001**	NA	NA	NA

### Auto- and heterotrophic ^2^H-fractionations in response to N fertilization and tissues

**Fig. 2.**
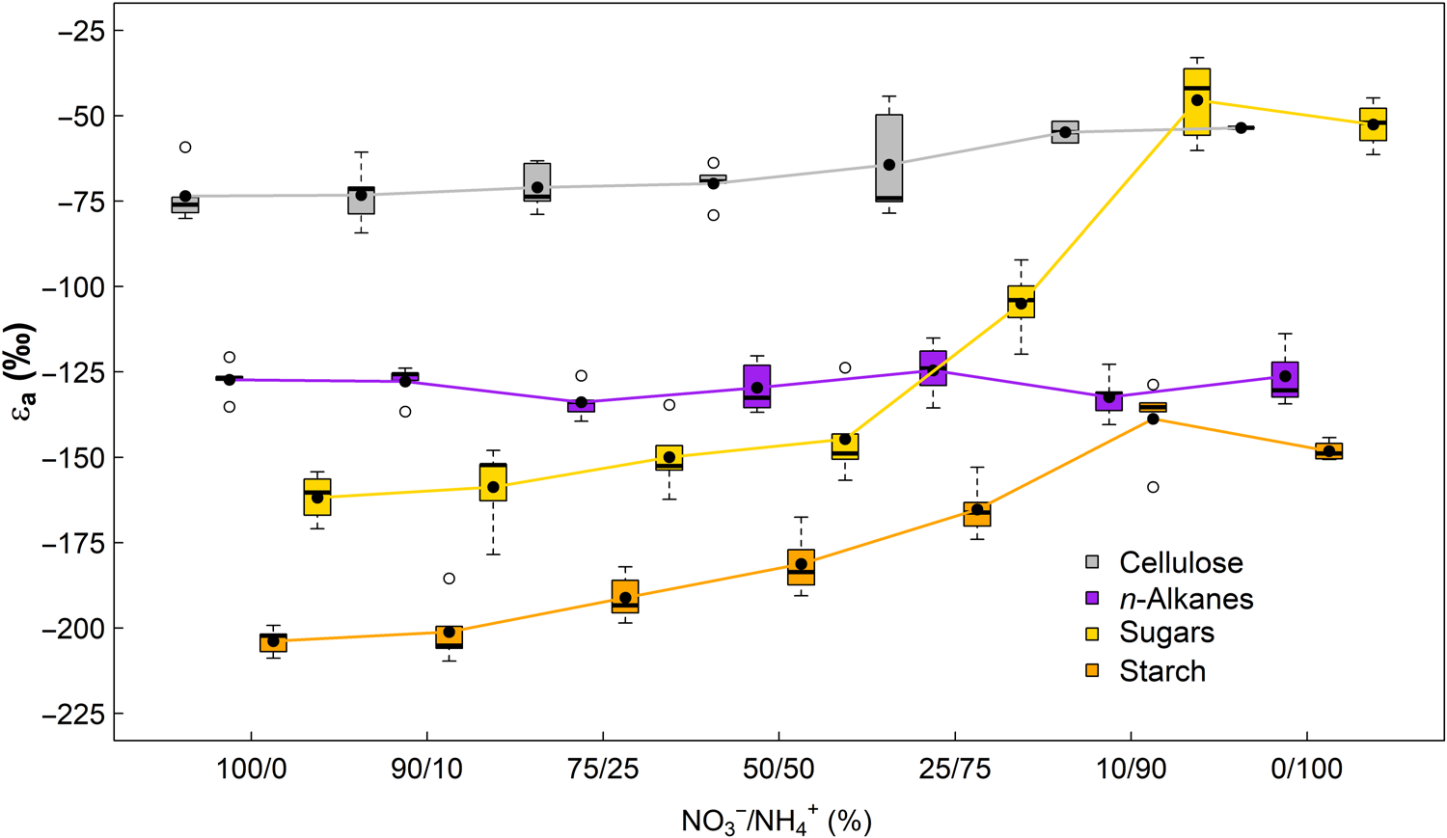
The apparent autotrophic ^2^H-fractionation factor (ε_a_) for compounds in tobacco (*N. sylvestris*) plants. ε_a_ values (= δ^2^H of leaf compound − δ^2^H of leaf water) for different carbohydrates and *n*-alkanes are shown. Plants were treated with 6 mM N fertilization solutions differing in their nitrate to ammonium ratio (NO_3_^−^/NH_4_^+^). ε_a_ of *n*-alkanes is based on the weighted δ^2^H mean of *n*-C_27_, *n*-C_29_, *n*-C_31_, and *n*-C_33_. Black dots indicate mean ε_a_ values. The boxes represent the median and the 25% upper/lower quartiles, while the tails represent the 10 and 90% limits of the data.

Because plant compounds were ^2^H-depleted compared to water (*P* < 0.001; Fig. 1), the apparent autotrophic ^2^H-fractionation factors (ε_a_, Eq. 1) were negative for all carbohydrates and *n*-alkanes. For carbohydrates, ε_a_ values increased with increasing NH_4_^+^ concentrations for sugar and starch (both *P* < 0.001) and cellulose (*P* = 0.025; Table 1 and Fig. 2). Cellulose mean ε_a_ values varied between −73.5 and −53.5‰, while mean ε_a_ values of sugars and starch ranged between −161.8 and −45.4‰, as well as −203.8 and −138.7‰, respectively. ε_a_ values of *n*-alkanes showed no response to N fertilization and averaged by −128.8‰ (*P* > 0.05; Table 1 and Fig. 2). Among all compounds, ε_a_ values of cellulose were the highest, followed by those of *n*-alkanes, sugars, and starch under NO_3_^−^-dominated conditions and those of sugars, *n*-alkanes, and starch under NH_4_^+^-dominated conditions. Sugar ε_a_ values under NH_4_^+^-dominated conditions were similar to those of cellulose of other N fertilization treatments (*P* > 0.05, Tukey post hoc test).

**Fig. 3.**
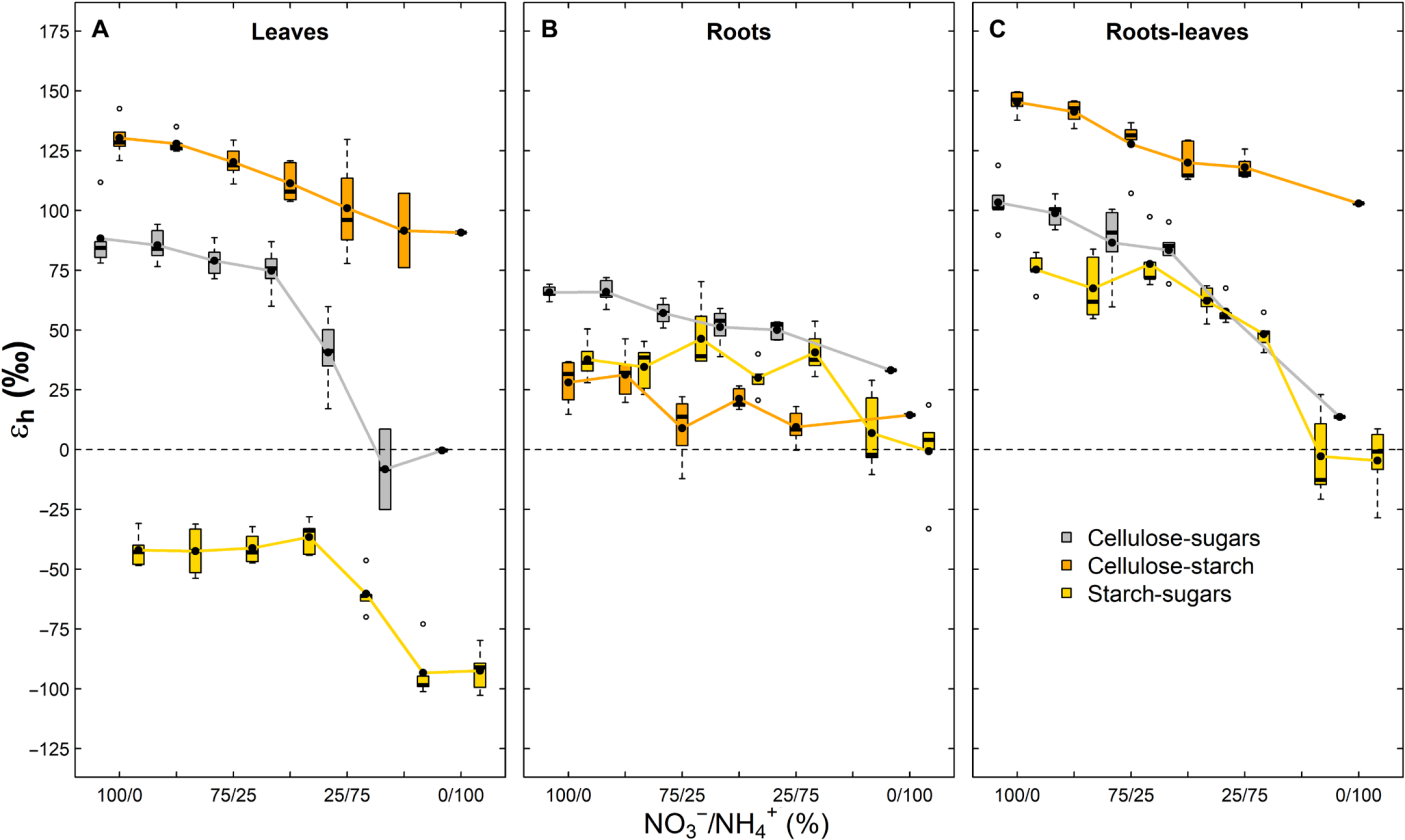
The apparent heterotrophic ^2^H-fractionation factor (ε_h_) among carbohydrates in tobacco (*N. sylvestris*) plants. ε_h_ values (= δ^2^H of compound A − δ^2^H of compound B) within (**A**) leaves, within (**B**) roots, and between (**C**) roots and leaves are shown. Plants were treated with 6 mM N fertilization solutions differing in their nitrate to ammonium ratio (NO3^−^/NH4^+^). The dashed line denotes ε_h_ = 0, while black dots indicate mean ε_h_ values. The boxes represent the median and the 25% upper/lower quartiles, while the tails represent the 10 and 90% limits of the data.

Apparent heterotrophic ^2^H-fractionations (ε_h,_ Eq_._ 2) among carbohydrates within and between leaves and roots decreased with increasing relative NH_4_^+^ concentrations (*P* < 0.01; Table 1 and Fig. 3), with overall larger variations in ε_h_ values of leaves than in roots. Along the N fertilization gradient, ε_h_ values between sugars and cellulose ranged from −0.3 to 88.3‰ within leaves and 33.2 to 66.0‰ within roots, with ε_h_ approaching 0‰ under NH_4_^+^-dominated conditions (Fig. 3, A and B). In comparison, ε_h_ values between starch and cellulose were generally higher and varied from 90.8 to 130.3‰ within leaves and 8.9 to 31.4‰ within roots. ε_h_ values between leaf sugars/starch and root cellulose approximately reflected the ε_h_ pattern observed within leaves (Fig. 3C). ε_h_ values between starch and sugars within leaves were negative and ranged from −93.3 to −36.6‰, while the same ε_h_ values within roots were generally positive and varied from −0.6 to 46.4‰ (Fig. 3, A and B). ε_h_ values between leaf sugars and root starch varied from −4.5 to 77.6‰ (Fig. 3C).

### Correlations among hydrogen isotopic parameters in the N fertilization experiment

Correlation analyses show that δ^2^H and ε_a_ values of all carbohydrates were generally positively related to each other in both plant tissues (*R* = 0.45 to 0.97; fig. S4) and to those in water (*R* = 0.41 to 0.81). ε_h_ values of all carbohydrates were negatively related to δ^2^H and ε_a_ values of any other compound and water (*R* = −0.37 to −0.97). In comparison, the relationship of δ^2^H of ε_a_ values of *n*-alkanes with carbohydrates and water in leaves was generally positive but weaker (*R* = 0.4 to 0.56) or not related, except for ε_a_ values of *n*-alkanes which were weakly negatively related to δ^2^H values of leaf water (*R* = −0.36). 

### Biophysical and biochemical drivers of hydrogen isotopes in plant compounds

A principal components analysis (PCA) of the combined hydrogen isotopic parameters of this study (i.e., δ^2^H, ε_a_, and ε_h_) with the biophysical and biochemical data of a previous study (*48*) reveals that the data of both plant tissues (Fig. 1) can be statistically separated into NO_3_^−^-dominated conditions (i.e., 100/0 to 50/50) and NH_4_^+^-dominated conditions (i.e., 25/75 to 0/100) (fig. S5). The PCA also revealed that hydrogen isotopic parameters of carbohydrates are related to various biophysical (e.g., net assimilation rates, *A*_net_; dark respiration rates, *R*_dark_) and biochemical traits (e.g., concentrations of sugars, S; concentrations of starch, ST) in leaves. Similar relationships for hydrogen isotope parameters of carbohydrates were also observed in roots, but the PCA pattern was slightly more scattered, and relationships become weaker or opposite [e.g., S, *R*_dark_, and nitrate reductase (NR)]. Hydrogen isotope parameters of *n*-alkanes (δ^2^H.na and εa.na) showed only weak correlations with lipid concentrations (L) and NSC in leaves.

**Fig. 4.**
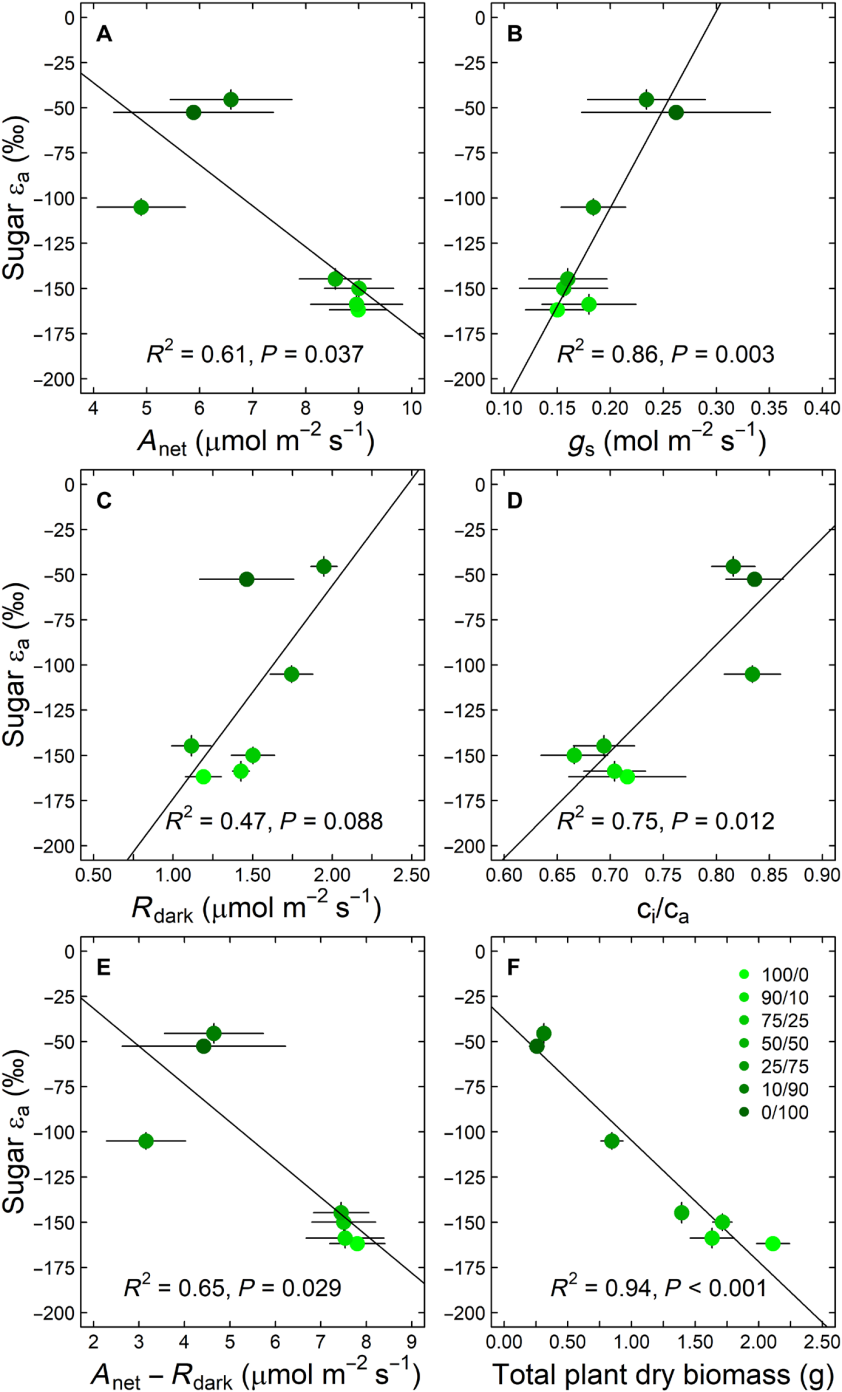
Biophysical drivers of ^2^H-fractionation in carbohydrates of tobacco (*N. sylvestris*) plants. The relationships between the apparent autotrophic ^2^H-fractionation factor for sugars (sugar ε_a_, δ^2^H of sugars − δ^2^H of leaf water) and plant biophysical traits are shown. Plants were treated with 6 mM N fertilization solutions differing in their nitrate to ammonium ratio (NO_3_^−^/NH_4_^+^). Black line indicates linear regression. (**A**) Net assimilation rate, *A*_net_; (**B**) stomatal conductance, *g*_s_; (**C**) leaf dark respiration rate, *R*_dark_; (**D**) ratio of intercellular and ambient CO_2_ concentrations, c_i_/c_a_; (**E**) difference between *A*_net_ and *R*_dark_; (**F**) total of leaf and root dry biomass. Mean values and SEs are shown.

**Fig. 5.**
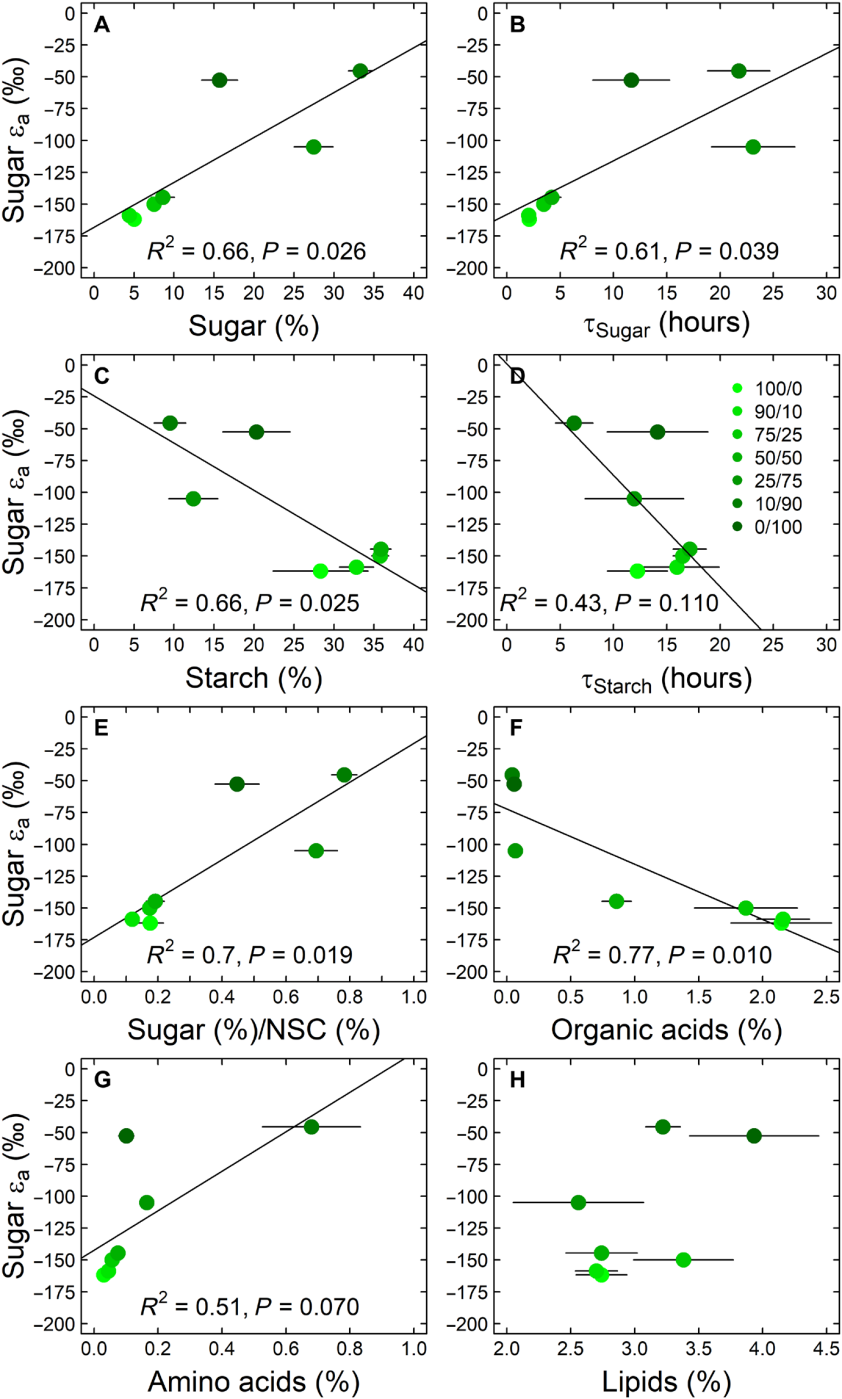
Biochemical drivers of ^2^H-fractionation in carbohydrates of tobacco (*N. sylvestris*) plants. The relationships between the apparent autotrophic ^2^H-fractionation factor for sugars (sugar ε_a_, δ^2^H of sugars − δ^2^H of leaf water) and biochemical traits in leaves of tobacco (*N. sylvestris*) plants are shown. Plants were treated with 6 mM N fertilization solutions differing in their nitrate to ammonium ratio (NO_3_^−^/NH_4_^+^). Black line indicates linear regression. (**A**) Sugar concentrations in percentage of dry leaf biomass and (**B**) their turnover time, τ_sugar_; (**C**) starch concentrations in percentage of dry leaf biomass and (**D**) their turnover time, τ_starch_; (**E**) proportion of sugars in total leaf NSC pool; (**F** and **G**) mean concentrations of highly abundant organic and AA in percentage of dry leaf biomass, respectively. (**H**) Concentration of weight-estimated total lipid fraction (*P* > 0.05). Mean values and SEs are shown.

Given the strong intercorrelation among the isotopic parameters (fig. S4), we selected ε_a_ of sugars as a general proxy for a detailed visualization of the biophysical and biochemical drivers of δ^2^H values of carbohydrates. For biophysical traits (Fig. 4), we found that ε_a_ of sugars was negatively related to *A*_net_ and the difference between *A*_net_ and *R*_dark_ (i.e., as a proxy for the respiratory loss per assimilated CO_2_) and positively to stomatal conductance *g*_s_, the ratio of intercellular and ambient CO_2_ concentrations, c_i_/c_a_ and *R*_dark_, with *R*^2^ values ranging between 0.47 and 0.86 (Fig. 4, A to E). Growth as indicated by total leaf and root dry biomass showed the strongest negative relationship with ε_a_ of sugars among all measured parameters (Fig. 4F). For biochemical traits, ε_a_ of sugars was negatively related to concentration of organic acid (OA), ST, and the turnover time of ST (τ_Starch_) and positively to S, ST/NSC, and the turnover time of S (τ_Sugar_), and AA, with *R*^2^ values ranging between 0.43 and 0.77 (Fig. 5, A to G). In contrast, ε_a_ of sugars was not related to L (Fig. 5H). Besides, ε_a_ of sugars was negatively related to traits of the N metabolism such as the ratio of carbon to nitrogen (C/N), the nitrogen isotope composition (δ^15^N), and NR (fig. S6).

### Comparing observed and modeled δ^2^H of cellulose in the N fertilization experiment

**Fig. 6.**
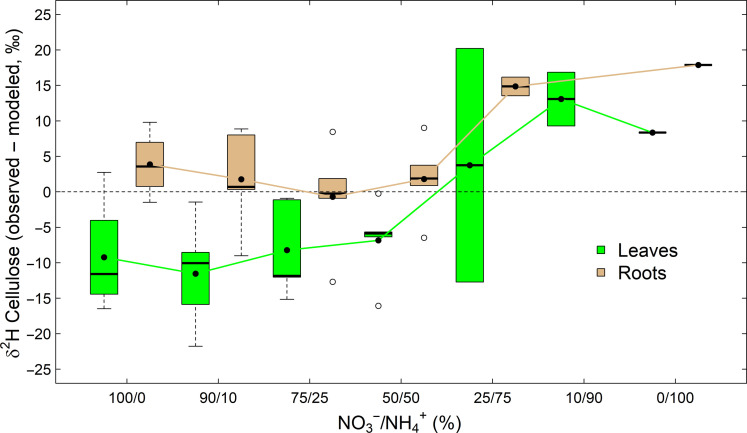
Modeling of the hydrogen isotopic composition (δ^2^H) of cellulose in tobacco (*N. sylvestris*) plants The differences between observed and modeled δ^2^H values of cellulose in leaves and roots are shown. Plants treated with 6 mM N fertilization solutions differing in their nitrate to ammonium ratio (NO_3_^−^/NH_4_^+^). The modeled δ^2^H values of cellulose are based on Eq. 3 (*15*). Dashed line denotes the zero line, while black dots indicate mean values. The boxes represent the median and the 25% upper/lower quartiles, while the tails represent the 10 and 90% limits of the data.

Subtracting the RE model outcome (Eq. 3) from the observed δ^2^H values of leaf and root cellulose shows that the model output deviated from the observed ones (Fig. 6). In leaves, observed δ^2^H values were up to 11.5‰ lower than modeled δ^2^H values under the NO_3_^−^-dominated conditions but up to 13.1‰ higher under the NH_4_^+^-dominated conditions. In roots, the difference between observed and modeled δ^2^H values were close to 0 under the NO_3_^−^-dominated conditions; however, observed δ^2^H values were up to 17.9‰ higher than the modeled δ^2^H values under the NH_4_^+^-dominated conditions. Thus, in most cases, the RE model outcome did not perfectly match the observed δ^2^H values of cellulose along the N fertilization gradient.

### δ^2^H values of compounds in wild type and starch-deficient mutant plants

**Fig. 7. F7:**
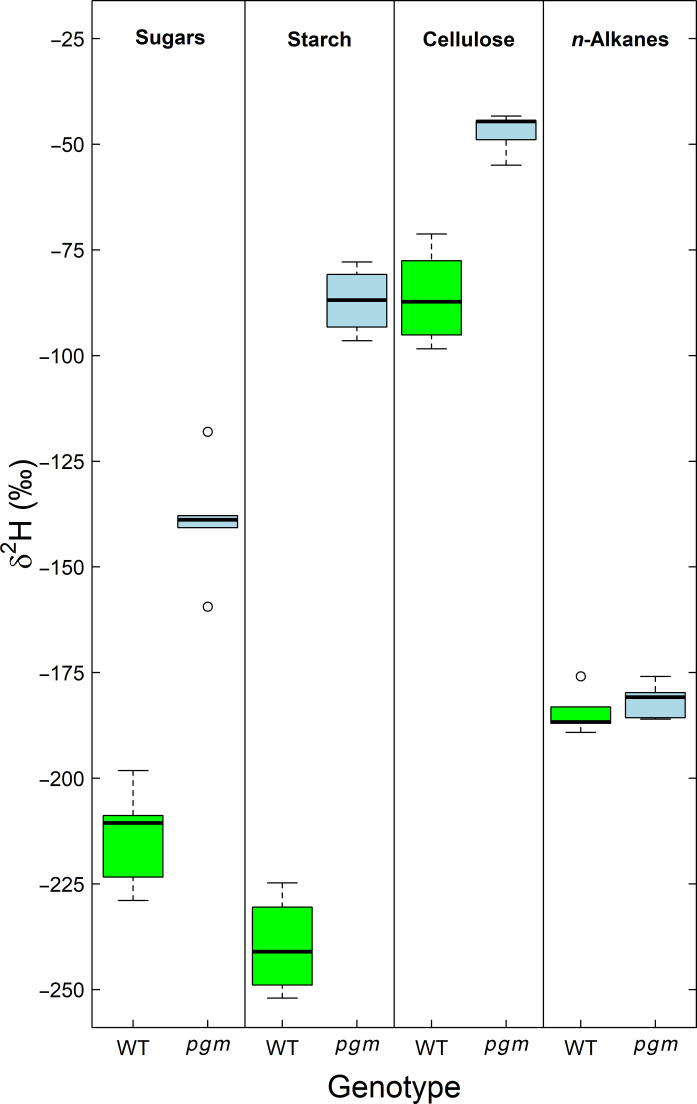
Starch deficiency effect on the hydrogen isotopic composition (δ^2^H) of compounds in tobacco (*N. sylvestris*) leaves. δ^2^H values of carbohydrates and *n*-alkanes in leaves of wild type (WT) and starch deficient *pgm* knockout mutant plants (*pgm*) are shown. δ^2^H of carbohydrates reflect the nonexchangeable carbon-bound hydrogen (δ^2^H_ne_, see Materials and Methods), while δ^2^H of *n*-alkanes represents the weighted mean of *n*-C_27_, *n*-C_29_, *n*-C_31_, and *n*-C_33_. The boxes represent the median and the 25% upper/lower quartiles, while the tails represent the 10 and 90% limits of the data.

Starch deficiency caused by the knockout of PGM in the mutant plant induced a ^2^H-enrichment in all carbohydrates of the *pgm* mutant compared to the WT plants (*P* < 0.001; Table 1 and Fig. 7), with the effect being higher for starch (152.7‰) and sugars (75.0‰) than for cellulose (38.7‰). In contrast and despite a tendency toward higher values in *pgm* compared to WT plants, the weighted average δ^2^H values of *n*-alkanes were not significantly affected by starch deficiency (*P* > 0.05[Fig F7]). Among all compounds, cellulose was the most ^2^H-enriched followed by *n*-alkanes, sugars, and starch in WT and by starch, sugars, and *n*-alkanes in *pgm* plants (*P* < 0.001). Besides, δ^15^N values of leaf organic matter were not significantly different between *pgm *(5.8 ± 1.0‰) and WT plants (4.4 ± 1.2‰, mean ± SD).

## DISCUSSION

### Identifying major drivers of δ^2^H variations in plant compounds

When examining the origin of the H atoms of carbohydrates and *n*-alkanes in plants, one can anticipate distinct sensitivities of the δ^2^H values in responses to treatments. Glucose molecules (i.e., as a reference for carbohydrates) have seven carbon-bound H atoms. A total of 14% and 43% of their H atoms are derived from NADPH and water, respectively, while the remaining 43% is derived from triose phosphate or photorespiratory processes. *n*-Alkanes, for example of 29 carbons, have 60 carbon-bound H atoms. A total of 47 and 24% of their H atoms are sourced by NADPH and water, respectively, while the remaining 29% of H atoms is derived from acetyl-CoA precursors and thus from sugar or photorespiratory processes (*7*, *9*). Thus, the way treatments affect plant water, NADPH, and/or precursor δ^2^H values should result in distinct isotopic responses among different plant compounds.

In the N treatment experiment, we observed only small δ^2^H variations in plant water (Table 1 and Fig. 1). The ^2^H-enrichment in leaf and root water under NH_4_^+^ compared NO_3_^−^ conditions is likely explained by the strong differences in growth along the N gradient (fig. S1). Smaller plants with a reduced root system under NH_4_^+^ conditions have likely taken up proportionally more topsoil water that experienced evaporative isotopic enrichment despite regularly watering. The larger plants with their well-distributed root system under NO_3_^−^ conditions took soil water from the whole pot (fig. S3). Because *g*_s_ was not affected by the N treatment effect (*48*), we assume that changes in the plant water flux, which often go along with changes in NO_3_^−^ / NH_4_^+^ ratios (*54*), did not modulate plant water isotopes in our study. In the starch deficiency experiment, we did not measure δ^2^H values of leaf water. However, both genotypes were grown under identical climatic conditions and exhibit no significant differences in the oxygen isotopic composition of leaf sugars and cellulose (fig. S7). Assuming a close relationship between carbohydrate and leaf water oxygen isotopic compositions (*55*, *56*), this finding, supported by a prior study (*9*), suggests that variations in leaf water isotopes are not a substantial contributing factor to the isotopic differences observed in plant compounds between *pgm* and WT plants in our investigation. Thus, the relatively low variation in the isotopic composition of plant water in both experiments suggests that the strong response in δ^2^H values of carbohydrates is related to other hydrogen metabolic sources such as NADPH and precursor molecules.

In this regard, it is interesting that the N treatment effect on δ^2^H values of *n*-alkanes fades after normalization with leaf water δ^2^H values (e.g., ε_a_ values; Table 1, Fig. 2, and fig. S8). This suggests that δ^2^H variations in leaf water solely can explain those of *n*-alkanes. This agrees with previous observations showing that *n*-alkanes can be used for the reconstruction of environmental δ^2^H values of water (*1*, *57*), while plant carbohydrate δ^2^H values appear driven by plant metabolic changes (*9*, *26*). Given that δ^2^H values of *n*-alkanes show little sensitivity to our treatments (Table 1 and Figs. 1 and 7) and that the H of *n*-alkanes is relatively more sourced by NADPH than H of sugars, this indicates that the δ^2^H values of the total NADPH pool (i.e., including photosynthetic and other sources of NADPH) are relatively constant along the N fertilization gradient. It is thus unlikely that isotopic variation in NADPH drives variations in δ^2^H values of carbohydrates. This is further supported by recent findings showing that the majority of variations observed in δ^2^H values of cellulose-derived glucose molecules can be explained by the glucose positions H_1_ and H_2_, which are likely not sourced by NADPH (*9*, *26*).

To some extent, the difference in treatment sensitivity among δ^2^H values of plant compounds could be explained by their timing of biosynthesis and turnover. While leaf sugars and starch are known to have a high turnover rate and reflect the most recent conditions, leaf cellulose and *n*-alkanes are known to be mainly produced early during the leaf development and to have low turnover rates and therefore a longer metabolic lifetime (*25*, *30*). Thus, while leaf assimilates can integrate the effects of the treatments late in the experiment, cellulose and *n*-alkanes are likely to have been already largely synthesized during the first 2 weeks, potentially with little treatment effect. While *n*-alkanes may undergo partial renewal during the growing season (*58*), our approximately 5-week treatment period may not have been sufficiently long to establish a distinct treatment effect on the δ^2^H values of *n*-alkanes (*59*). However, the δ^2^H response of plant compounds to the starch deficiency treatment, which was genetically induced and therefore consistently expressed, closely resembled that observed in the N fertilization experiment (Fig. 7). This implies that the δ^2^H values of plant compounds in this study are unlikely to be influenced by substantial temporal variations in the magnitude of the treatments. Overall, if we exclude plant water, NADPH sources, and temporal influence of biosynthesis time as major sources of H isotopic variations, then our data indicate that the relatively large variations in plant carbohydrate δ^2^H values are likely driven by the isotopic composition of precursor molecules that have experienced both different ^2^H-fractionations and different levels of H isotope exchange with plant water in their metabolic pathways.

From a biochemical perspective, sugars and transitory starch function as precursors for the biosynthesis of plant compounds, and thus, one can expect that changes in the relative abundance of sugar and starch concentrations in leaves might be cause of δ^2^H treatment sensitivity of cellulose. Our results prove that changes in relative abundances of leaf sugar and leaf starch are directly affecting the ^2^H-fractionation during cellulose biosynthesis in both experiments. This implies a strong relationship between ε_a_ values of leaf sugars in the N fertilization experiment of our study compared to sugar and starch concentrations, as well as their turnover times, of a previous study (Fig. 5, A to D). These relationships hold when sugar concentrations are normalized with the total NSC concentrations (Fig. 5E). Consequently, a ^2^H-enrichment in all carbohydrates under NH_4_^+^ conditions compared to NO_3_^−^ conditions concurs with increasing sugar and decreasing starch concentration. This is consistent with a ^2^H-enrichment in carbohydrates in response to starch deficiency (Table 1 and Fig. 7), where the lack of starch is compensated by higher sugar concentrations in *pgm* compared to WT plants (*49*). These findings align with the recent hypothesis proposing that an elevated relative sugar concentration within the NSC pool results in a ^2^H-enrichment in carbohydrates. This is due to the increased likelihood of hydrogen isotope exchange reactions between carbon-bound H and plant water, such as those involving isomerase reactions affecting triose or hexose phosphate in various biochemical pathways (*7*). This metabolic effect on δ^2^H values, related to the relative contribution of sugar and starch to the total NSC pool, might be ecologically relevant under water limited conditions (*24*, *26*) that often lead to higher sugar and lower starch concentrations in leaves following osmotic adjustments (*60*). On the other hand, the change in the δ^2^H values and concentrations of sugars and starch did not affect δ^2^H values of *n*-alkanes in both experiments (Table 1, Figs. 1 and 7, and fig. S5). This is supported by a study demonstrating that *n*-alkanes show indeed a much weaker δ^2^H response than cellulose to different levels of starch deficiency in different species (including the *N. sylvestris* *pgm* mutant) (*9*). Likely, the original δ^2^H signal of the photosynthesis-derived triose phosphates, which carries the signature of the treatment, is only weakly modified before incorporation into carbohydrates. In contrast, triose phosphates that are supplied via acetyl-CoA to the acetogenic pathway experience more modifications before lipid synthesis, potentially through a postmalonate H isotope exchange with water (*61*).

Furthermore and along the N fertilization experiment, we observed that ε_a_ values of leaf sugars of our study were related to decreasing OA and increasing AA concentrations but not to lipid concentrations (Fig. 5, F to H), with the concentration data (except for lipids) being derived from a previous study (*48*). This result goes along with the starch deficiency experiment, where a decrease in OA concentrations (*49*) matches with ^2^H-enrichment in plant carbohydrates in *pgm* compared to WT plants (Fig. 7). In tobacco plants, the OA concentrations reflect mainly the concentration of malate and citrate (*48*). These OA are known to function as carbon skeletons for anaplerotic reactions connected to the citric acid cycle and subsequently for synthesis of AA of the aspartate and glutamate family, getting more enhanced under stress conditions. Carbon skeletons are particularly needed for the fixation of NH_4_^+^ (via the glutamine synthetase and glutamine oxoglutarate aminotransferase/glutamate synthase pathway), which can function as a N source under reduced NO_3_^−^ availability but also to remove toxic NH_4_^+^ from plant tissues. This might also explain the observed negative correlation between ε_a_ values of leaf sugars and indicators of N metabolism, such as and C/N ratios, NR activity, and δ^15^N values (fig. S6). While the latter could suggest a potential linkage between ^15^N- and ^2^H-fractionations through N metabolism, it is crucial to note that this correlation may have been simply induced by the δ^15^N values of the supplied N species and that δ^15^N values in leaf organic matter were not affected by starch deficiency. It should also be noted that the relative abundance of AA, OA, and lipids were one magnitude smaller compared to NSC in the N fertilization experiment (fig. S2). These compound pools may thus represent a minor source of variations in δ^2^H values compared to sugars and starch.

Our results of the N treatment experiment also provide evidence that the change in the relative abundance of plant compounds and its consequences for hydrogen isotope patterns in plants is strongly interlinked with various biophysical traits (Fig. 4), which function as indicators of plant performance and were deduced from a previous study performing the same experiment but focusing on carbon isotope fractionation (*48*). We found that tobacco plants having higher relative starch than sugar concentrations under high NO_3_^−^ treatments performed well, as indicated by biomass production and *A*_net_ being at the highest (Fig. 4, A and F, and fig. S1), while *g*_s_, *R*_dark_, and c_i_/c_a_ were at the lowest (Fig. 4, B to D). In contrast, tobacco plants having a lower relative starch than sugar concentrations under NH_4_^+^-dominated treatments performed less. Processing NH_4_^+^ and its inherent toxicity reduced growth and *A*_net_ while increasing *R*_dark_ to maintain the basic metabolic functions (*48*, *62*). The change in the biophysical traits were tightly correlated to ε_a_ values of leaf sugars, which is reflected by a ^2^H-depletion in NO_3_^−^ and ^2^H-enrichment in NH_4_^+^-dominated treatments (Fig. 4, A to D). This finding aligns with the ^2^H-enrichment in plant carbohydrates in *pgm* compared to WT plants (Fig. 7), with the former being described to have lower *A*_net_ and growth rates but higher c_i_/c_a_ (*49*, *63*). ε_a_ values of leaf sugars were negatively correlated with difference between *A*_net_ and *R*_dark_ (Fig. 4E). This suggests that under elevated stress conditions and declining plant performance, increased respiratory processes relative to assimilation rates may preferentially respire isotopically light assimilates. This would result in a ^2^H-enrichment in the remaining substrates. Alternatively, the positive relationship between *R*_dark_ and ε_a_ values of leaf sugars might be explained by an increased throughput of sugars through futile cycles that increases the likelihood of H-exchange with water (*6*, *64*) or by relative changes in the contribution of plant respiratory compounds and associated isotope fractionation (*48*). In summary, our results demonstrate that biochemical and biophysical traits function as drivers of hydrogen isotope patterns in plant carbohydrates.

### On the isotopic difference between leaf and root assimilates

Through the application of an improved water vapor equilibration technique for determining the isotopic values of nonexchangeable H atoms of carbohydrates (*31*), this study provides exceptional data describing the differences in δ^2^H values of sugars and starch in leaves and roots (Figs. 1 and 3). In leaves, starch was up to 40‰ ^2^H-depleted compared to sugars in both experiments and across all treatments, except for the minor leaf starch residues in *pgm* mutant plants that are likely produced via a cytosolic bypass reaction (*49*). The ^2^H-depletion of transitory starch agrees with previous observations and can be explained by an expressed isotope fractionation due to the kinetic ^2^H-isotope effect on the plastidic phosphoglucose isomerase (EC 5.3.1.9)–catalyzed reaction leading to ^2^H-depletion in glucose-6-phosphate used for starch biosynthesis (*31*, *46*). In contrast, starch in roots was about 40 to 90‰ ^2^H-enriched compared to sugars in both plant tissues under NO_3_^−^-dominated conditions and compared to leaf starch across all N fertilization treatments. Besides, we also observed a ^2^H-enrichment in root sugars compared to leaf sugars under NO_3_^−^-dominated conditions. We suggest that the mechanistic reason for the ^2^H-enrichment in root starch and sugars compared to leaf assimilates can be explained in a similar manner as the heterotrophic ^2^H-enrichment in cellulose, where the leaf-level signal is progressively lost due to H isotope exchange of intermediates with water or through additional ^2^H-fractionations in the metabolic pathway.

The ^2^H-enrichment observed in root starch supports recent hypotheses proposing that heterotrophic starch, which functions as a long-term carbon storage or reserves, exhibits higher ^2^H-enrichment when compared to fresh assimilates (*28*). For instance, seeds (which typically have a relatively high starch content) have been found to be strongly ^2^H-enriched compared to water-soluble fraction and dry matter in other tissues of wheat plants (*35*). A ^2^H-enrichment was also observed in early compared to late wood (*47*) and during the early phase of leaf development for cellulose (*30*) and lipids (*25*). Thus, in cases where the supply of fresh assimilates is low and ^2^H-enriched reserve of starch are proportionally more used for growth or synthesis of metabolites (*29*), this may lead to an overall ^2^H-enrichment of the biomass. Under stress conditions, this could be amplified by biochemical and biophysical effects and lead to further ^2^H-enrichment of the plant biomass. In such cases, an intramolecular analysis of sugars would be needed to distinguish the sources of δ^2^H variations (*26*).

### Toward an improved mechanistic isotope model of cellulose δ^2^H values

The mechanisms described above have implications for hydrogen isotope models. For instance, the RE model (Eq. 3) (*14*, *15*, *51*) assumes that variations in carbohydrates δ^2^H values are solely determined by those in leaf and source water by using constant theoretical ^2^H-fractionation factors (i.e., ε_a*_ = −171‰, ε_h*_ = 158‰) and a constant proportion of H isotope exchange with source water (*f* = 0.36). Our experiments suggest otherwise and show that the RE model overestimates carbohydrate δ^2^H values in leaves under NO_3_^−^-dominated conditions and underestimates them in leaves and roots under NH_4_^+^-dominated conditions (Fig. 6). We also observed that the RE model performed better for roots under the less stressful NO_3_^−^-dominated conditions (Fig. 6), suggesting that the RE model is most effective in heterotrophic tissues when plant performance is high (e.g., under good growth conditions). This is consistent with δ^2^H values in tree-ring cellulose being correlated with δ^2^H of source water under well-watered experimental conditions (*15*) or with δ^2^H of precipitation on large geospatial scales (*21*).

Disentangling the sources of the mismatches between the RE model and measured data remains challenging. The low δ^2^H variations across plant water in our study cannot fully explain isotopic variations in leaf and tree-ring cellulose (Fig. 1), suggesting that mismatches are probably caused by δ^2^H variations in plant assimilates induced by biophysical and biochemical drivers. For instance, considering dynamic ε_a_ values of sugars and starch (Fig. 2) rather than a constant theoretical value of −171‰ could already improve the poor model outcome for temporal variations in tree rings (*14*). This would be also consistent with recent studies demonstrating that δ^2^H values of plant cellulose are primarily determined by ε_a_ values of assimilates (*6*). Besides, we noticed that ε_h_ values became lower in leaves and roots under less stressful conditions (Fig. 3). This suggests that the effects occurring in heterotrophic sink cells, which modify the original leaf-level δ^2^H values of sugars before cellulose synthesis, may also have changed along the N fertilization gradient. Estimations of RE model parameters through rearranging Eq. 3 show that *f* and ε_h*_ values are indeed changing along the N treatment but that the treatment effect on the model parameters is much more severe if, instead of a constant ε_a*_ value, dynamic ε_a_ values of sugars of our study are considered (fig. S9). While constant ε_a*_ value led to minor variations in *f* (0.28 to 0.43) with increasing values under NH_4_^+^-dominated conditions for leaves and roots, dynamic ε_a_ values lead to much higher variations in *f* (−0.17 to 0.39) with decreasing values under NH_4_^+^-dominated conditions for leaves and roots. Thus, in the cases of *f* values close to 0 (i.e., no H isotope exchange with source water), δ^2^H values of cellulose of low-performing plants under severe stress conditions can theoretically be explained solely by responses of leaf-level ^2^H-fractionations. However, the observations made in our experiments do not allow to disentangle whether it is the *f* or ε_h*_ that changed or both. It should also be considered that we only worked with tobacco plants in this study. Yet, a recent study demonstrated that δ^2^H values of carbohydrates across a large set of tree and shrub species show distinct phylogenetic differences (*40*). Consequently, cellulose isotope models may include order-, family-, or genera-specific ^2^H-fractionation factors if comparisons are made across different species.

### Implications

δ^2^H values of plant carbohydrates show a dynamic response to nitrogen fertilization with different ratios of NO_3_^−^/NH_4_^+^ and to starch deficiency, which was expressed as a ^2^H-enrichment with increasing stress conditions (i.e., WT versus *pgm *or NO_3_^−^ versus NH_4_^+^). We explain this pattern mechanistically through changes in biophysical (e.g., *A*_net_ and *R*_dark_) and biochemical (e.g., sugar or starch concentration in the NSC pool) traits. Because these traits are well-known to respond to environmental conditions such as relative humidity (RH), soil moisture, temperature, and CO_2_ concentrations, δ^2^H values of plant carbohydrates could function as a valuable environmental proxy if further developed (*7*). Therefore, further studies are certainly needed to test whether our findings are valid across different species, environmental conditions, and geological archives (e.g., tree rings or plant remains in peatlands, lake sediments, and ice caves).

Furthermore, the differences in δ^2^H values between ^2^H-enriched stored versus ^2^H-depleted fresh assimilates, as elucidated in our study, offer potential means of distinguishing the metabolic origins of carbon skeletons involved in the biosynthesis of compounds. This differentiation could extend to fundamental plant functions, encompassing growth, responses to both abiotic and biotic stress, as well as maintenance and growth respiration, under various environmental influences. These ideas are supported by recent studies suggesting δ^2^H values of plant compounds as a potential proxy for tracing changes in carbon use strategies and carbon allocation in response to climatic or nonclimatic impacts (*28*, *29*) or to identify the carbon source of soil organic matter (*65*, *66*). The large δ^2^H variation in plant assimilates in our study (>100 ‰) might also function as a selection trait for plant functional responses to stress conditions in breeding or garden experiments.

Certainly, our study has implications for hydrogen isotope models, as we show that isotopic variations in leaf and source water are not sufficient to explain variations in cellulose δ^2^H values under all conditions. Considering the influence of biophysical and biochemical drivers on ^2^H-fractionation shaping plant carbohydrates will be key for improving tree-ring model outcomes (*24*) and will lead to more accurate predictions of climatic conditions (e.g., RH or temperature) and water sources (*2*, *67*).

Our results also show that *n*-alkanes δ^2^H values were not influenced by both the biochemical and biophysical processes connected to nitrogen fertilization (with different ratios of NO_3_^−^/NH_4_^+^) and starch deficiency. This should reinforce the confidence that can be put in δ^2^H values of acetogenic lipids for reconstructing (paleo-)hydroclimatic conditions. Last, this study is a rare example illustrating that the measurements of δ^2^H values on diverse compound classes (e.g., sugars, starch, cellulose, and lipids) can help elucidate underlying processes of ^2^H-fractionation in plants (*9*).

## MATERIALS AND METHODS

### Plant material

#### Nitrogen (N) fertilization experiment

For this experiment, we have grown tobacco plants (*N. sylvestris*) from seeds in climate chambers at ETH Zurich and treated them with a N fertilization gradient following a previous study (*48*). Briefly, we transplanted one 2-week-old seedling, originally grown on an organic substrate, into each 750-ml pot containing sterile sandy substrate and perlite (5:1). We prepared N free nutrient solution (*68*) and added NO_3_^−^/NH_4_^+^ in the percent ratios of 100/0, 90/10, 75/25, 50/50, 25/75/, 10/90, and 0/100. We applied the treatments to five individuals (*N* = 5) by watering them every other day, alternating between the prepared N fertilization solutions and deionized water, to ensure a consistent water supply. The pH of drained soil solutions ranged between 6.5 in 100/0 and 5 in 0/100 treatments. The growth conditions were 12-hour light with a photosynthetic photon flux density (PPFD) at plant height of ~400 μmol m^−2^ s^−1^, 22/20°C (day/night) temperatures, and constantly ~60% RH.

After 5 weeks of treatment (equivalent to 7 weeks of growth), we harvested the plant material within 3 hours in the middle of the light period. First, we sampled fully developed leaves and transferred them to 12-ml gas-tight glass vials (“exetainer,” Labco, Lampeter, UK), cut the plants at the root crown, and transferred the residue of the above-ground biomass to paper bags. Second, we freed the root material from sand by washing with tap water and dried it with paper towels. We transferred the coarse roots (diameter > 2 mm, including the root crown) to 12-ml exetainer vials and fine roots (<2 mm) to paper bags. Third, soil samples covering the complete soil profile were transferred to 12-ml exetainer vials. All samples in exetainer and paper bags were frozen directly in liquid nitrogen and stored at −20°C. Samples in exetainer vials were designated for water extraction, while those in paper bags were freeze-dried and kept at a dry place until further use. The isotopic composition of irrigation water was monitored by taking samples of N fertilization solutions and deionized water over the course of the growing period (δ^2^H = −79.3 ± 0.9‰, δ^18^O = −11.2 ± 0.3‰; mean ± SD). For analysis of the isotopic composition of water vapor (δ^2^H = −140.0 ± 4.4‰, δ^18^O = −19.8 ± 0.2‰), samples of the climate chamber air were trapped several times in the week before the harvest by pumping air through a U-tube in ethanol/dry ice slurry (*69*).

While the presented data on isotopic composition, biomass, and percentage of lipids (%) are based on the above-described experiment, we further deduced data of biophysical and biochemical traits from previous experiments with the same experimental design as described above with methods described therein (*48*). This includes compound concentrations (i.e., sugars, starch, AA, and OA; fig. S2), gas exchange [i.e., assimilation (*A*_net_), dark respiration rates (*R*_dark_), stomatal conductance (*g*_s_), activity of NR, C/N ratio, and δ^15^N]. C/N and δ^15^N values in leaves and roots, as well as root-based data of compound concentrations and NR, have not been published so far. Concentrations of sugars (S), AA, and OA are presented as sum of individual compounds due to their very similar N fertilization response (glucose, fructose, and sucrose for S; alanine, asparagine, glutamine, and serine for AA; citrate and malate for OA). NSC reflects the sum of total sugar and starch concentrations. The turnover time of sugars (τ_sugar_) and starch (τ_starch_) is calculated by dividing the total sugar or starch concentrations by *A*_net_, respectively, and by using an inferred connection between leaf area and dry biomass. Strong relationships between δ^2^H values of organic matter and carbohydrates along the N treatment between the previous and this study, as well as for dry biomass, demonstrate that the results of the two studies are comparable and reproducible (figs. S4 and S10).

#### Starch deficiency experiment

For this experiment, we have taken plant material of experiment no. 3 described in a previous study (*49*). Briefly, we have grown tobacco (*N. sylvestris*) WT and *pgm* knockout mutant plants from seeds in a climate chamber. Two weeks after germination on organic substrates, we transplanted five individuals per genotype (*N* = 5) to 1-liter pots with standard potting soil. After 6 weeks in pots (equivalent to 8 weeks of growth), we collected leaf material following 4 hours of light exposure and promptly transferred them to paper bags. Subsequently, all samples were immediately frozen in liquid nitrogen. The material was then stored frozen at −20°C, subjected to freeze-drying, and kept in a dry location until further use. The growth conditions were 12-hour light with a PPFD at plant height of ~160 μmol m^−2^ s^−1^, 22/18°C (day/night) temperatures, and constantly 70% RH.

### Water extraction and isotope analyses

For the extraction of plant and soil water by cryogenic vacuum distillation (CVD), we followed the method and setup described in (*70*). We observed an average ^2^H-depletion of 6.7‰ in root compared to soil water but no difference in oxygen isotopes (fig. S3). The difference is likely explained by methodological ^2^H-fractionation during extraction of water from heterotrophic tissues (*53*). We determined the δ^2^H and δ^18^O values of the CVD-extracted water and the liquid water samples of the vapor traps by a thermal conversion/elemental analyzer that was coupled via a ConFlo III reference interface to an isotope ratio mass spectrometer Delta^plus^XP at ETH Zurich (TC/EA-IRMS, all supported by Finnigan MAT, Bremen, DE) (*71*). The precision of quality control standards was 0.6‰ for δ^2^H and 0.1‰ for δ^18^O and data is presented on the international Vienna Standard Mean Ocean Water (VSMOW) scale. 

### Plant carbohydrate extraction and isotope analyses

For the N fertilization experiment and to achieve enough biomass of all treatments, we pooled the dry plant material from the exetainers after water extraction and paper bags. We then estimated the total leaf and root biomass (fig. S1). For both experiments, half of the material was ball-milled to fine powder (MM400, Retsch, Haan, DE), while the other half was cut in small pieces.

We extracted NSCs in form of sugars and starch from ~100-mg powdered leaf and root material, if not restricted by biomass availability (fig. S1). Hot deionized water (1.5 ml) was added to plant material, and the water-soluble compounds were extracted at 85°C for 30 min in a thermos-shaker. Subsequently, the supernatant was separated from the pellet after centrifugation (10,000*g*, 2 min), and the sugars (“Sugars”; i.e., mixture of mainly fructose, glucose, sucrose, and sugar alcohols) were extracted through purification with a commercially available cartridge system (OnGuard II A, H and P, Dionex, Sunnyvale, CA, USA) (*72*). Starch was extracted from the remaining pellet after several wash steps with deionized water and ethanol, enzymatic digestion with a heat-stable α-amylase at 85°C for 2 hours, and purification of mono- and oligosaccharides of starch with centrifugation filters (*73*). Aliquots of sugars (~0.5 mg) and starch-derived sugars (~1 mg) solutions were then transferred into 5.5 mm by 9 mm silver foil capsules (Ag; IVA Analysentechnik, Meerbusch, DE), frozen at −20°C, and subsequently freeze-dried. The Ag capsules were then folded and packed into a second set of Ag capsules of the same size to avoid loss of sugar material during hot water vapor equilibration (*31*) and kept in a desiccator until further treatment.

Cellulose, or more precisely holocellullose, was extracted from 25 to 40 mg of leaf and root pieces, with the exact amount depending on the available biomass (fig. S1). The plant pieces were packed in filter bags (Ankom Technology, Macedon, NY, USA) and transferred to an Erlenmeyer beaker. The bags with the sample material were treated twice with 5% NaOH for 2 hours at 60°C to remove lipids, tannins, and hemicellulose. Afterward, the samples were extensively washed with cold deionized water and bleached several times with 7% NaClO_2_ (pH 4 to 5) for 10 hours at 60°C. The cellulose powder was then dried, taken out of the filter bag, rewetted with deionized water in a reaction tube, homogenized with ultrasonic transducer (UP200S, Hielscher, Teltow, Germany), and freeze-dried (*55*). Cellulose samples of ~1 mg were packed into 3.3 mm by 5 mm Ag capsules (IVA Analysentechnik, Meerbusch, DE) and kept in a desiccator until further steps.

The measurement of the nonexchangeable δ^2^H (δ^2^H_ne_) values of sugars, starch, and cellulose samples followed an improved water vapor equilibration method (*31*). Briefly, the exchangeable hydrogen atoms of each carbohydrate sample were equilibrated with water vapor of two different isotopic compositions (δ^2^H_w1_ = −160‰, δ^2^H_w2_ = −428‰) in a metal chamber at 130°C and subsequently dried with N_2_ gas. The samples were then immediately transferred to an autosampler (“Costech Zero Blank”, NC Technologies, Milano, Italy), which was closed and directly vacuumed to avoid any adsorption of atmospheric water traces or isotope exchange with ambient water vapor. δ^2^H of the equilibrated samples (δ^2^H_e1_ and δ^2^H_e2_) were then measured with TC/EA-IRMS system (also Finnigan MAT) at WSL Birmensdorf.

δ^2^H_e1_ values were first offset corrected with δ^2^H values of IAEA-CH-7 polyethylene foil (PEF), and, where required, an amount correction was applied. Then, the %-proportion of exchanged hydrogen during the equilibrations (*x*_e_) was calculated on the basis of δ^2^H_e_ and δ^2^H_w_ values

$$x_{e}=\frac{\delta^{2}H_{e1}-\delta^{2}H_{e2}\;}{\alpha_{e-w}\cdot \left(\delta^{2}H_{w1}-\delta^{2}H_{w2}\right)}$$ (4)

where α_e-w_ is the fractionation factor of 1.082 for cellulose (*74*, *75*), which we considered to be similar for sugars (*31*). δ^2^H_ne_ can then be calculated using the results of one of the two analyses (e.g., δ^2^H_e1_ and δ^2^H_w1_)

$$\delta^{2}H_{\text{ne}}=\frac{\delta^{2}H_{e1}-x_{e}\cdot \alpha_{e-w}\cdot \delta^{2}H_{w1}-1000\cdot x_{e}\cdot (\alpha_{e-w}-1)\;}{1-x_{e}}$$ (5)

δ^2^H_ne_ of plant carbohydrates (indicated as δ^2^H values throughout the manuscript) were then brought to the international VSMOW scale using previously calibrated δ^2^H_ne_ values of in-house sucrose and cellulose standards (*31*). Mean *x*_e_ values of PEF, sucrose, and cellulose standards were 0.0, 0.35, and 0.21, respectively. δ^2^H values of the different compounds were measured with a typical precision (SD) of 2 to 4‰.

### Leaf *n*-alkane extraction and isotope analyses

We extracted lipids from ~200 mg of powdered material according to a published protocol (*76*), if not restricted by biomass availability (fig. S1). Extraction of *n*-alkanes of roots was not perused due to insufficient amount of biomass. Briefly, ultrasonic extraction was performed with dichloromethane/methanol (93:7, v/v). Weighed air-dried extracts were redissolved in hexane, and aliphatic hydrocarbons including mainly *n*-alkanes were separated from other lipids by elution over activated silica in a Pasteur pipette. After air-drying, the aliphatic hydrocarbon fractions were redissolved and transferred to autosampler vials for gas chromatographic measurements. Compositional screening of *n*-alkane composition was achieved on an Agilent 7890 gas chromatograph (GC) equipped with a multimode inlet and a flame ionization detector. Identification was ensured by comparison of retention times with known standard substances. Thereafter, fractions were measured for compound-specific δ^2^H values of individual *n*-alkanes using a Trace 1320 GC (Thermo Fisher Scientific, Waltham, Massachusetts, USA) equipped with a programmable temperature vaporizer inlet. The GC was connected to a Delta V Plus isotope ratio mass spectrometer via GC-Isolink II and Conflo IV (all Thermo Fisher Scientific). Individual compounds were converted to H_2_ in the high-temperature conversion reactor of the GC-Isolink at 1420°C. The isotope ratio mass spectrometer was calibrated for δ^2^H values using certified *n*-alkane standard mixtures (A7 and B5; Schimmelmann Research, Indiana University Bloomington, IN, USA). Compound separation was achieved in both GC systems on a J&W DB-5MS column (50-m long, 0.2-mm inner diameter, 0.32-μm film thickness). One microliter of the fraction was injected at an inlet temperature of 80°C and kept isothermal for 0.5 min. Thereafter, the inlet temperature was ramped to 400°C at a rate of 800°C/min. The oven was kept isothermal at 70°C for 2 min and then ramped to 320°C at a rate of 5°C/min. The final temperature was held for 30 min. Precision (SD) of the δ^2^H values of individual *n*-alkanes was typically better than 2‰ after triplicate analyses and data is presented on the VSMOW scale. Given that the δ^2^H response to the N fertilization and starch deficiency treatments was generally low or not significant for all measurable *n*-alkanes (C_27,_ C_29,_ C_31,_ and C_33_; figs. S8 and S11), we received a proportion-normalized δ^2^H value for the *n*-alkane fraction. We therefore calculated weighted averages of *n*-alkane δ^2^H values by summarizing the fractions of the individual *n*-alkanes multiplied with their respective isotope values.

### Statistics

Measurements and analyses were typically performed on five individuals per N fertilization treatment or genotype (*N* = 5) in the current and previous studies (*48*, *49*). Because of low amounts of leaf and root biomass under high NH_4_^+^ conditions (10/90 and 0/100; fig. S1), none or only up to three individuals were analyzed for δ^2^H of cellulose and *n*-alkanes. We applied one-way or two-way analysis of variance (ANOVA) and Tukey post hoc tests to test for significant δ^2^H differences between the effects of tissues (i.e., leaves/roots) or compounds (i.e., water, carbohydrates, and *n*-alkanes) with effects of N fertilization or starch deficiency (WT versus *pgm*). The *t*-tests were applied to test for starch deficiency on the isotopic composition of plant compounds. A PCA was performed to illustrate the most important traits driving δ^2^H variations in plant compounds and water across the N fertilization gradient. First, data were gap-filled with the median of each parameter yet separately for NO_3_^−^ (i.e., 100/0 to 50/50) and NH_4_^+^ conditions (50/50 to 0/100) considering large differences for the majority of traits between the two treatment conditions (*48*). We then calculated mean values for each treatment considering that trait and isotope data were derived from different batches of plants. Last, we established separate data matrices for leaves and roots and performed a PCA with the *prcomp* function in stats package of base R. Variables were centered and scaled for standardization. Selected principal components had eigenvalues ≥ 1 and explained together ≥78% of the data. PCA was visualized with package factoextra (*77*). All statistical analyses and figures were performed and produced using R version 4.2.0. (*78*).
