# Supplementary material for: Biochemical and biophysical drivers of the hydrogen isotopic composition of carbohydrates and acetogenic lipids
Source: Sci Adv. 2024 Jul 10;10(28):eadl3591. doi: 10.1126/sciadv.adl3591 (PMC11235168; doi:10.1126/sciadv.adl3591)
Supplement: Supplementary file 1 — Figs. S1 to S11 [file sciadv.adl3591_sm.pdf]

Science Advances
AAAS

Supplementary Materials for

Biochemical and biophysical drivers of the hydrogen isotopic composition of carbohydrates and acetogenic lipids

Marco M. Lehmann *et al.*

Corresponding author: Marco M. Lehmann, marco.lehmann@wsl.ch;
Marc-André Cormier, ma_cormier@alumni.ethz.ch

Sci. Adv. **10**, eadl3591 (2024)
DOI: 10.1126/sciadv.adl3591

This PDF file includes:

Figs. S1 to S11

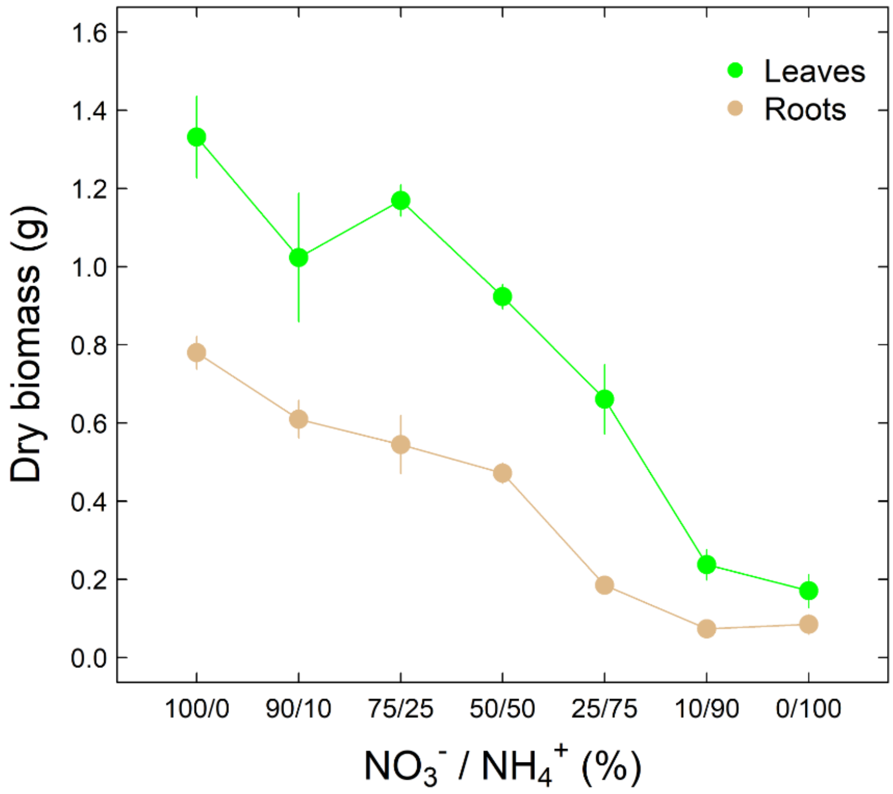

Figure S1: Leaf and root dry biomass of tobacco (*N. sylvestris*) plants treated with 6 mM N fertilization solutions differing in their nitrate to ammonium ratio (NO_3^-/NH_4^+). Mean values ± 1 SE are shown.

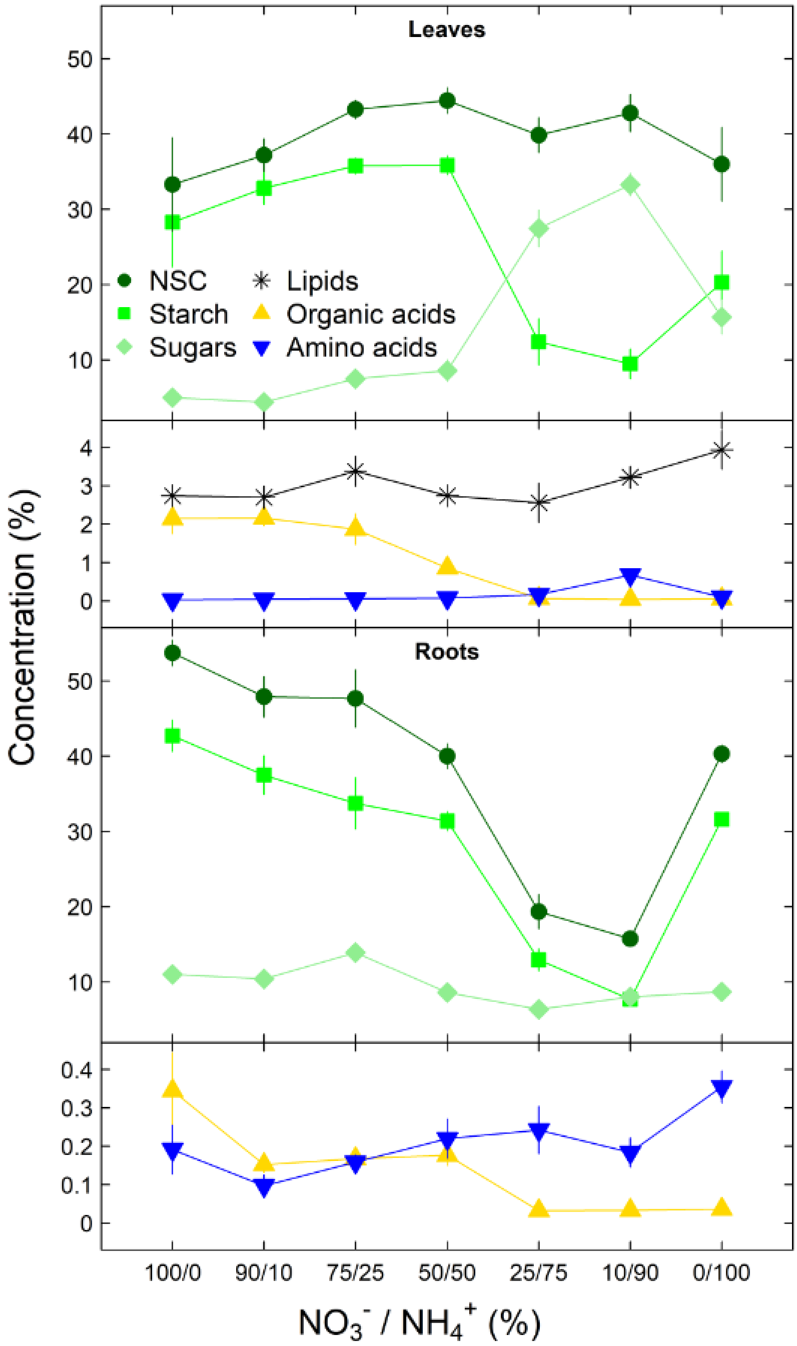

Figure S2: Concentration of different compounds in leaf and roots of tobacco (*N. sylvestris*) plants treated with 6 mM N fertilization solutions differing in their nitrate to ammonium ratio (NO_3^-/NH_4^+). Total lipid concentration in leaves is dry-weight based and were measured in this study. All other data is derived from experiments described in (*47*). Sugar concentration is the sum of fructose, glucose and sucrose, while non-structural carbohydrate (NSC) concentration is the sum of sugar and starch concentrations; Organic acid concentration is the sum of malate and citrate concentrations; Amino acid concentration is the sum of alanine, asparagine, serine, and glutamine concentrations. Mean values ± 1 SE are shown.

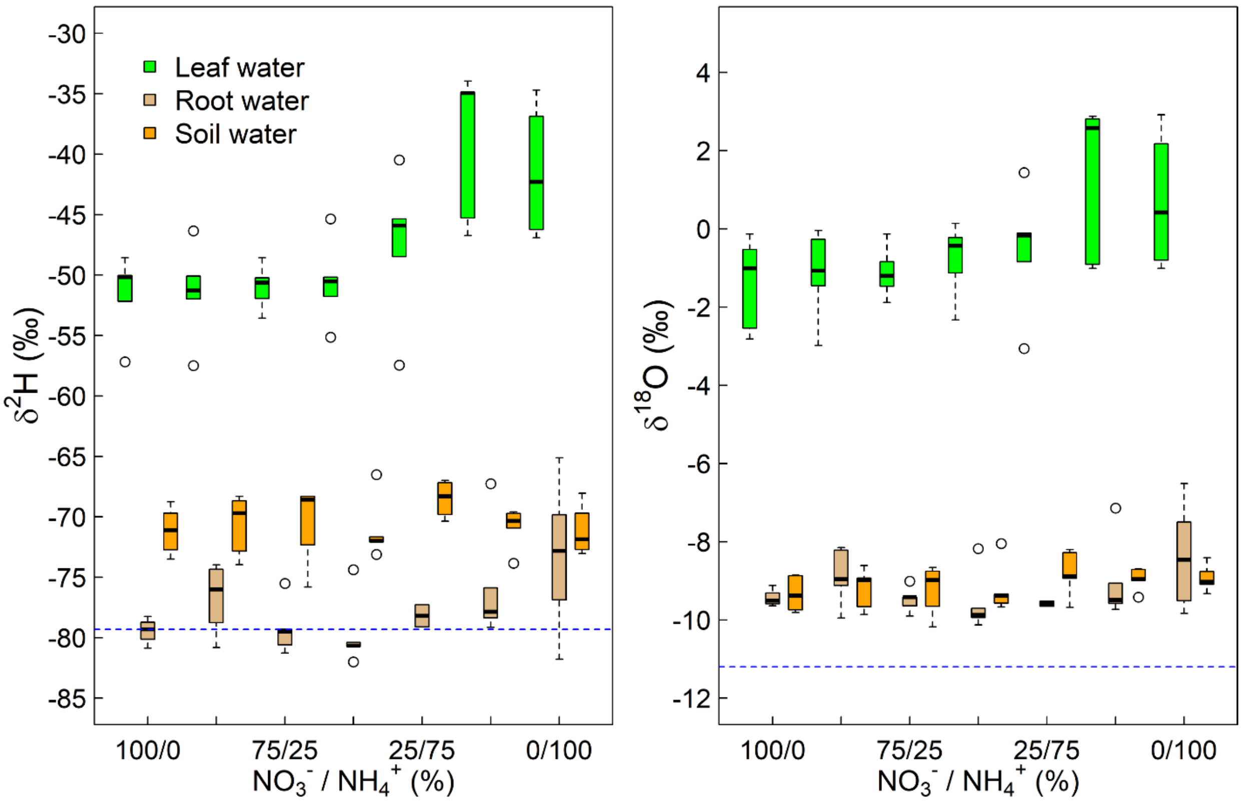

Figure S3: Hydrogen (δ^2H) and oxygen ($\delta^{18}O$) isotopic composition in soil water and in leaf and root water of tobacco (*N. sylvestris*) plants treated with 6 mM N fertilization solutions differing in their nitrate to ammonium ratio (NO_3^-/NH_4^+). Blue dashed line indicates mean δ^2H values of irrigation water during the experiment. The boxes represent the median and the 25% upper/lower quartiles, while the tails represent the 10% and 90% limits of the data.

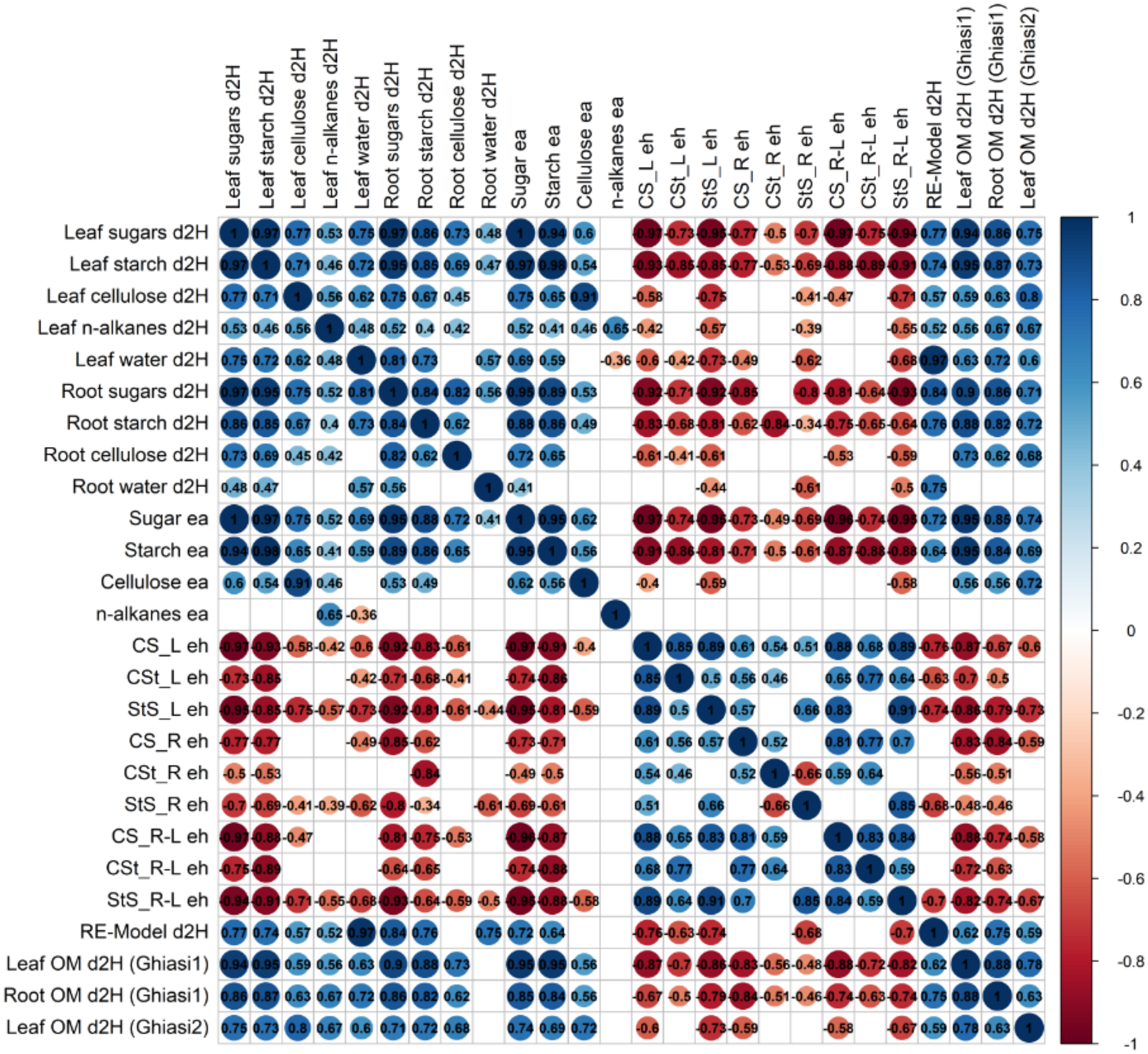

Figure S4: Correlation matrix of isotope parameters derived from the tobacco (*N. sylvestris*) plants treated with 6 mM N fertilization solutions differing in their nitrate to ammonium ratio (NO_3^-/NH_4^+). The matrix includes measured δ^2H (= d2H) values of water, carbohydrates (i.e., sugars (S), starch (ST), and cellulose (C)), *n*-alkanes, and organic matter (OM) in leaves and roots, modeled δ^2H values of cellulose (RE-model, Eq. 3), autotrophic ^2H-fractionations (ea), heterotrophic ^2H-fractionations among carbohydrates (eh). eh values are differentiated between C and S (CS), C and ST (CSt), St and S (StS) for roots (R), leaves (L) or between roots and leaves (R-L). δ^2H of carbohydrates and OM reflect the non-exchangeable carbon-bound hydrogen (δ^2H_{ne}, see methods), while δ^2H of *n*-alkanes represents the weighted average of *n*-C_{27}, *n*-C_{29}, *n*-C_{31}, *n*-C_{33}. Ghiasi1 and Ghiasi2 denotes δ^2H data of OM derived from the growing set 1 and 2 of Ghiasi *et al.* (ref #48), respectively. Pearson`s correlation coefficient (R) is given only for significant correlations ($P > 0.05$). Color legend illustrates strength of correlation. Correlation is based on data of individual plants (up to 5 per treatment).

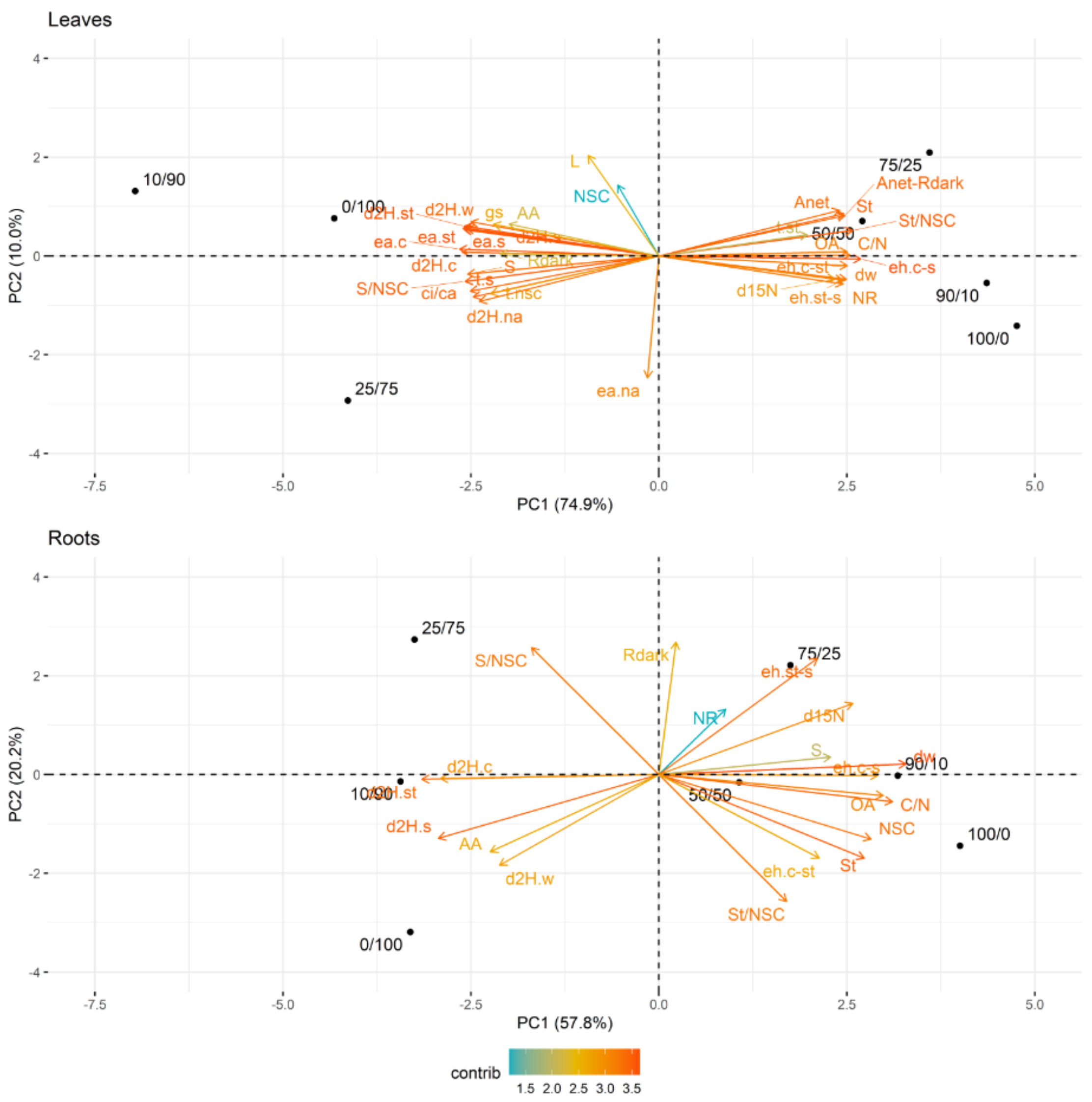

Figure S5: Principal component analysis of tobacco (*N. sylvestris*) leaves (top) and roots (bottom) treated with 6 mM N fertilization solutions differing in their nitrate to ammonium ratio (NO_3^-/NH_4^+). Treatments are shown as black points, while isotopic, biophysical, and biochemical traits are indicated by arrows. Color and lengths of arrows denote their contribution to the principal component and their importance to the individual treatments. δ^2H of sugars (d2H.s), starch (d2H.st), and cellulose (d2H.c) reflect the non-exchangeable carbon-bound hydrogen (δ^2H_{ne}, see methods), while δ^2H of *n*-alkanes (d2H.na) represents the weighted average of *n*-C_{27}, *n*-C_{29}, *n*-C_{31}, *n*-C_{33}. Further traits: δ^2H of water (d2H.w); $\delta^{15}N$ (= d15N) of organic matter; autotrophic ^2H-fractionation for sugars (ea.s), starch (ea.st), cellulose (ea.c), *n*-alkanes (ea.na); heterotrophic ^2H-fractionation between cellulose and sugars (eh.c-s), cellulose and starch (eh.c-st), starch and sugars (eh.st-s); sugar concentrations (S); starch concentrations (St); non-structural carbohydrate concentrations (NSC); St/NSC ratios (St/NSC); turnover time of S (t.s), St, (t.st) and NSC (t.nsc); total lipid concentrations (L); organic acid concentrations (OA); amino acid concentrations (AA); nitrate reductase (NR); net assimilation rate (Anet); dark respiration (Rdark); the difference between Anet and Rdark (Anet-Rdark); stomatal conductance (gs); ratio of intercellular to ambient CO_2 concentration (ci/ca); tissue dry weight (dw); carbon/nitrogen ratio (C/N).

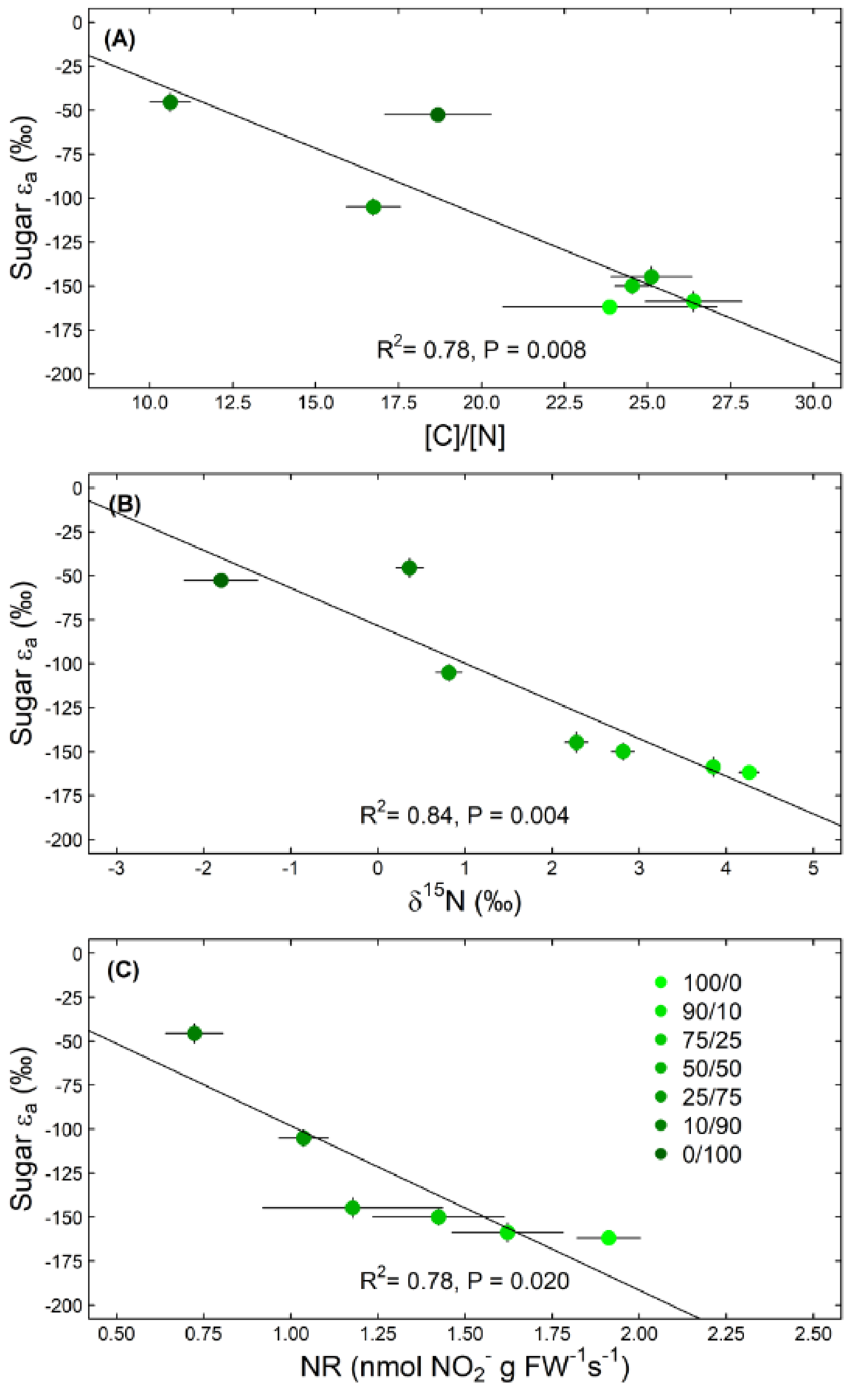

Figure S6: Relationship between autotrophic ^{2}H-fractionation factor (ε_a) of sugars and leaf traits related to nitrogen (N) metabolism in tobacco (*N. sylvestris*) plants treated with 6 mM N fertilization solutions differing in their nitrate to ammonium ratio (NO_3^-/NH_4^+). Black line indicates linear regression. [C]/[N] = ratio of carbon and N concentrations, $\delta^{15}N$ = nitrogen isotopic composition, NR = nitrate reductase activity. Mean values ± 1 SE are shown.

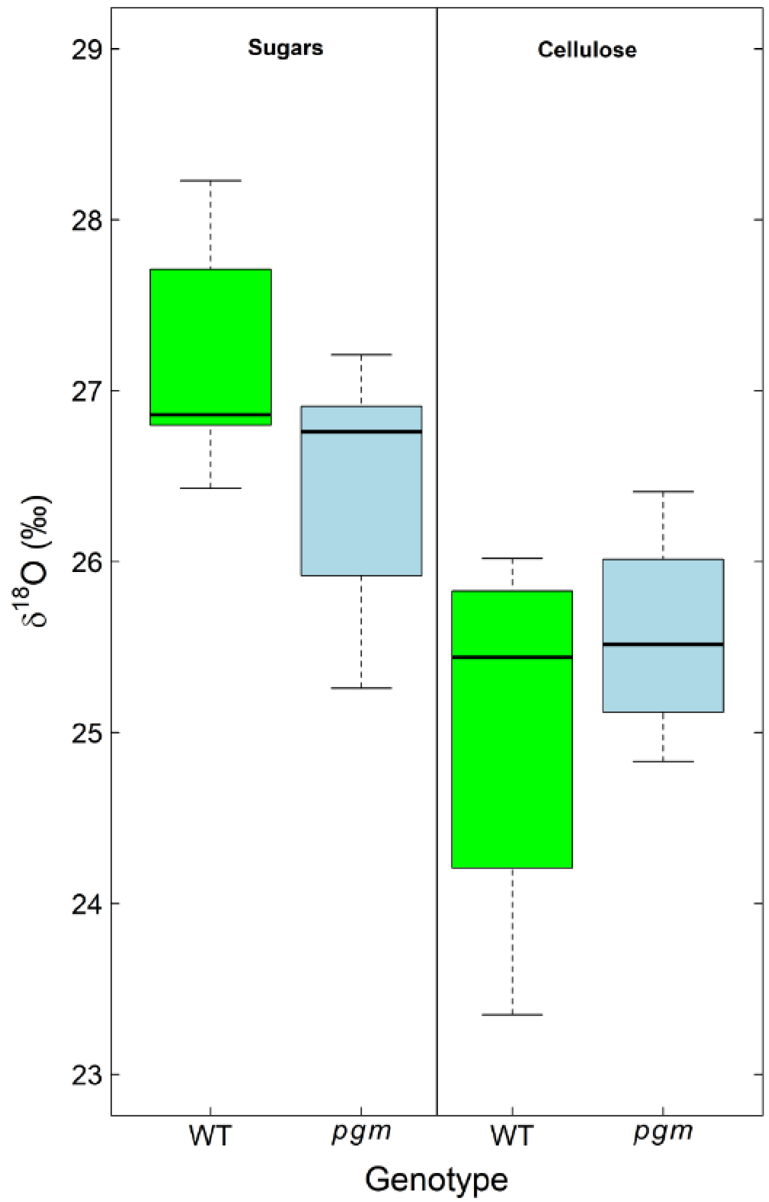

Figure S7: Oxygen isotopic composition ($\delta^{18}O$) of sugars and cellulose in tobacco (*N. sylvestris*) leaves of wild type (WT) and starch deficient phosphoglucomutase (*pgm*) knockout mutant plants. Starch deficiency had no effect on $\delta^{18}O$ values of both plant carbohydrates ($P > 0.05$, t-Test). The boxes represent the median and the 25% upper/lower quartiles, while the tails represent the 10% and 90% limits of the data.

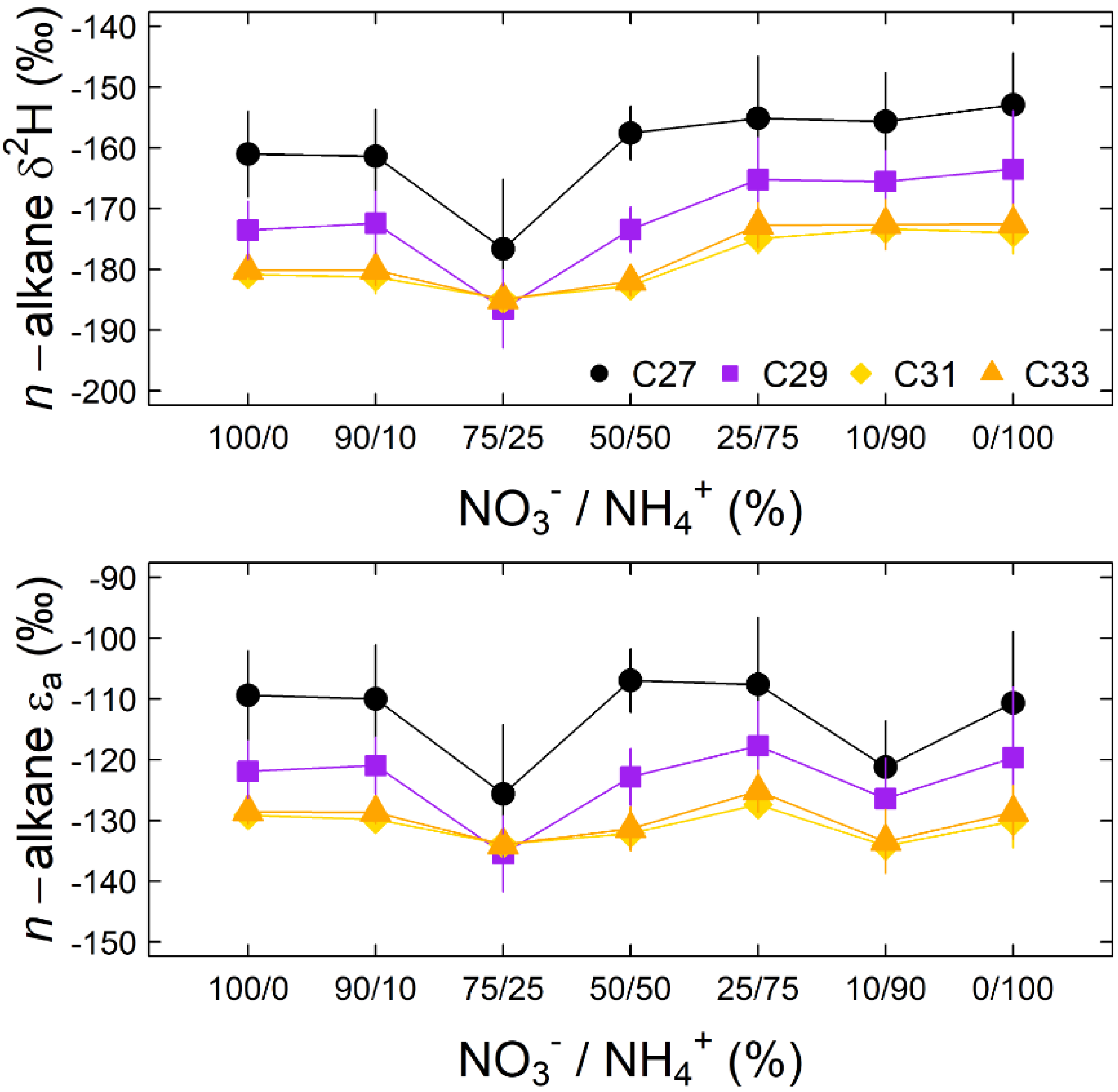

Figure S8: Hydrogen (δ^2H) isotopic composition in *n*-alkanes of different chain length of tobacco (*N. sylvestris*) plants treated with 6 mM N fertilization solutions differing in their nitrate to ammonium ratio (NO_3^-/NH_4^+). The lower panel shows the autotrophic ^2H fractionation factors of *n*-alkanes (ε_a, δ^2H of *n*-alkanes - δ^2H of leaf water). Mean values ± 1 SE are shown.

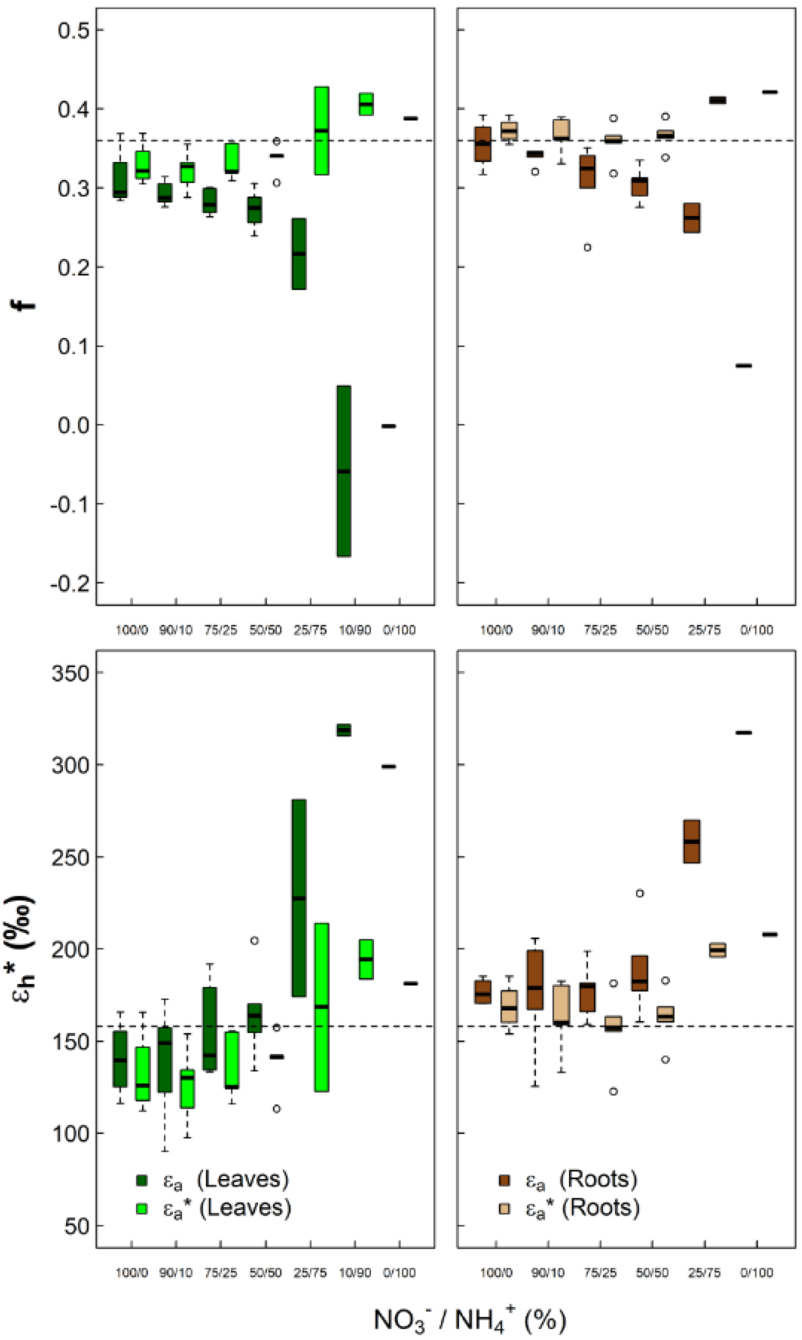

Figure S9: RE-model parameter estimation following rearranged Eq. 3. The proportion of H isotope exchange with source water (f) before cellulose biosynthesis and the theoretical heterotrophic ^{2}H fractionation factor (ε_{h^*}) are shown. Results denote two model scenarios using (1) a constant ε_{a^*} of -171 ‰ (ε_{a^*}) or (2) a dynamic ε_a of sugars (ε_a) for leaves and roots of this study. Tobacco (*N. sylvestris*) plants were treated with 6 mM N fertilization solutions differing in their nitrate to ammonium ratio (NO_3^-/NH_4^+). Dashed line indicates the literature value for f = 0.36 and ε_{h^*} = 158‰, respectively. Boxes represent the median and the 25% upper/lower quartiles, while the tails represent the 10% and 90% limits of the data.

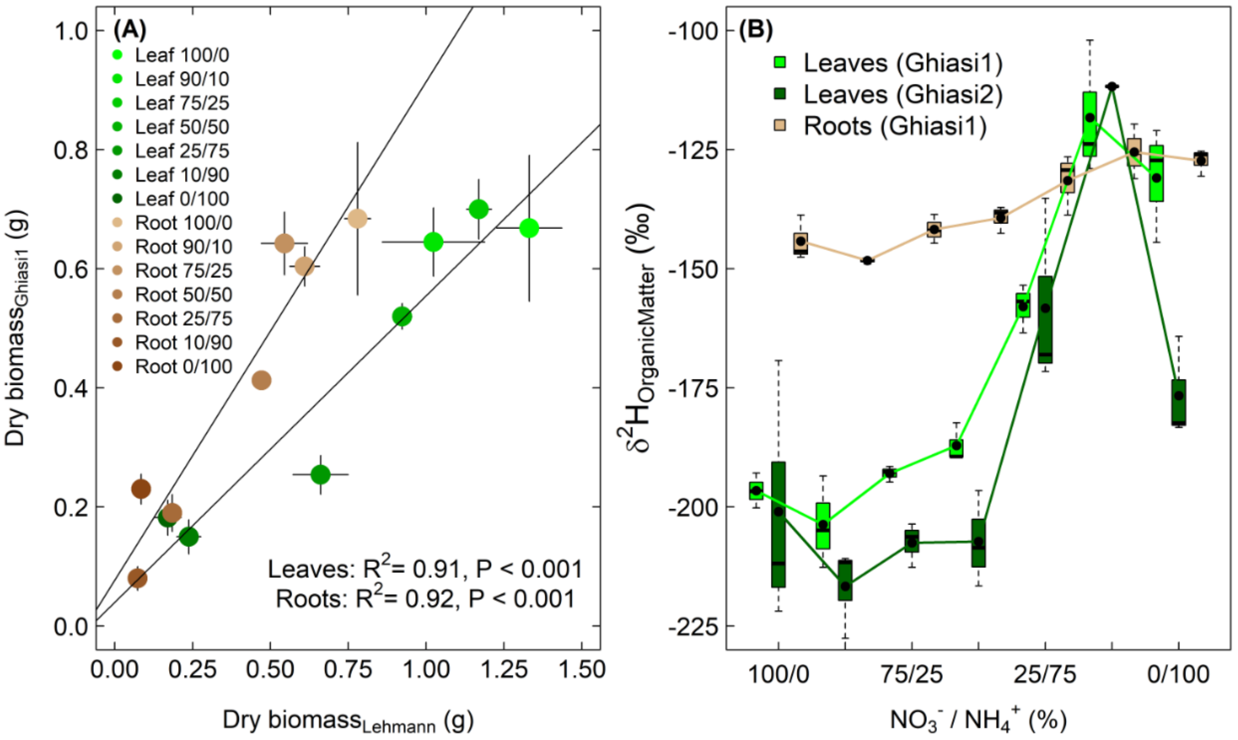

Figure S10: Dry plant biomass and the hydrogen isotopic composition of organic matter ($\delta^2H_{OrganicMatter}$) of tobacco (*N. sylvestris*) plants treated with 6 mM N fertilization solutions differing in their nitrate to ammonium ratio (NO_3^-/NH_4^+) derived from Ghiasi *et al.* (ref #48), (A) The relationship between the dry leaf and root biomass of Ghiasi *et al.* (ref #48) (Dry Biomass$_{Ghiasi1}$) and this study (Dry Biomass$_{Lehmann}$). Black line indicates linear regression. Mean values ± 1 SE are shown (N = 4-5). (B) $\delta^2H_{OrganicMatter}$ of leaf and root material of Ghiasi *et al.* (ref #48). Black dots indicate mean values. Boxes represent the median and the 25% upper/lower quartiles, while the tails represent the 10% and 90% limits of the data (N = 2-3). Ghiasi1 and Ghiasi2 denote δ^2H of OM derived from the growing set 1 and 2 of Ghiasi *et al.* (ref #48), respectively. The experimental conditions were the same between Ghiasi *et al.* (ref #48) and this study.

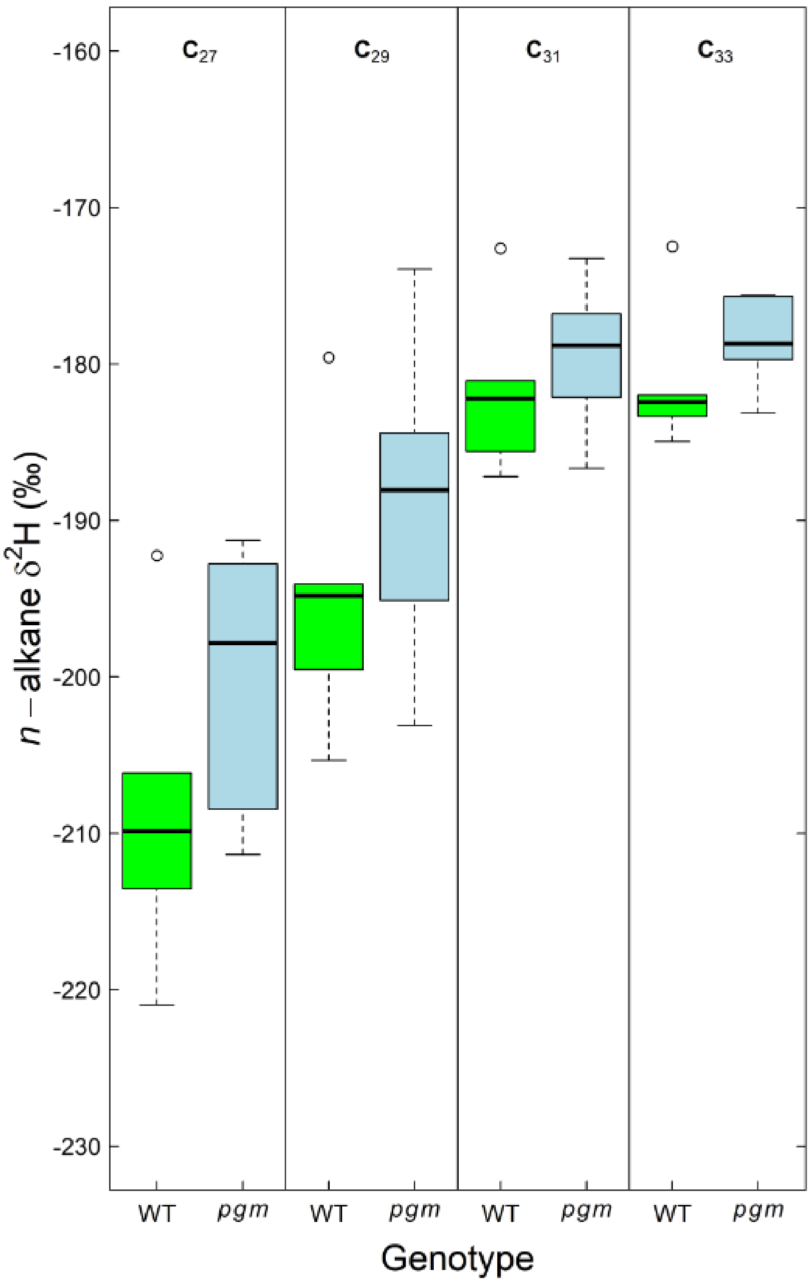

Figure S11: Hydrogen (δ^2H) isotopic composition of *n*-alkanes of different chain length of tobacco (*N. sylvestris*) leaves of wild type (WT) and starch deficient phosphoglucomutase (*pgm*) knockout mutant plants. Starch deficiency had no effect on the δ^2H values of *n*-alkanes of any chain length ($P > 0.05$, t-Test). The boxes represent the median and the 25% upper/lower quartiles, while the tails represent the 10% and 90% limits of the data.
